# Design and Assessment of Species-Level qPCR Primers Targeting Comammox

**DOI:** 10.3389/fmicb.2019.00036

**Published:** 2019-01-31

**Authors:** Natalie K. Beach, Daniel R. Noguera

**Affiliations:** Department of Civil and Environmental Engineering, University of Wisconsin–Madison, Madison, WI, United States

**Keywords:** comammox, *Nitrospira*, nitrification, low dissolved oxygen, biological nutrient removal, qPCR, real-time PCR, PCR primers

## Abstract

Published PCR primers targeting the ammonia monooxygenase gene (*amoA*) were applied to samples from activated sludge systems operated with low dissolved oxygen (DO) to quantify total and clade-level *Nitrospira* that perform complete ammonium oxidation (comammox); however, we found these existing primers resulted in significant artifact-associated non-target amplification. This not only overestimated comammox *amoA* copies but also resulted in numerous false positive detections in the environmental samples tested, as confirmed by gel electrophoresis. Therefore, instead of attempting to quantify comammox diversity, we focused on accurately quantifying the candidate comammox species. We designed specific and sensitive primers targeting 3 candidate species: *Candidatus* (*Ca.*) Nitrospira nitrosa, *Ca.* N. inopinata, and *Ca.* N. nitrificans. The primers were tested with *amoA* templates of these candidate species and used to quantify comammox at the species level in low DO activated sludge systems. We found that comammox related to *Ca.* N. nitrosa were present and abundant in the majority of samples from low DO bioreactors and were not detected in samples from a high DO system. In addition, the greatest abundance of *Ca.* N. nitrosa was found in bioreactors operated with a long solids retention time. *Ca.* N. inopinata and *Ca.* N. nitrificans were only detected sporadically in these samples, indicating a minor role of these comammox in nitrification under low DO conditions.

## Introduction

The oxidation of ammonium via nitrite to nitrate (i.e., nitrification) was historically considered a two-step process completed by phylogenetically distinct ammonia oxidizers and nitrite oxidizers. Although the complete oxidation of ammonium to nitrate by a single organism was theoretically possible, no microorganism with the ability to carry out both steps had been identified until late 2015 (Daims et al., 2015; van Kessel et al., 2015). These complete ammonium oxidizing (comammox) bacteria belong to the genus *Nitrospira*, which was known to contain only nitrite-oxidizing bacteria (NOB); therefore, an entire group of microorganisms with the ability to oxidize ammonia had been disguised for years (Daims et al., 2015; van Kessel et al., 2015). The discovery of comammox bacteria has redefined a key component of the global nitrogen cycle, initiating a renewed focus of recent nitrogen cycling research in environmental biotechnology (Chao et al., 2016; Palomo et al., 2016; Pinto et al., 2016; Stadler and Love, 2016; Bartelme et al., 2017; Fowler et al., 2017; Hu and He, 2017).

Within the wastewater treatment industry, the application of nitrification under very low dissolved oxygen (DO) concentrations (below 0.2 mg O_2_/L) is an exciting energy-saving approach to biological nutrient removal (BNR) (Park and Noguera, 2004; Paredes et al., 2007; Fitzgerald et al., 2015). Previous attempts to identify the key microorganisms responsible for nitrification in low DO bioreactors have been inconclusive, with the presence of known ammonia oxidizing bacteria (AOB) and ammonia oxidizing archaea (AOA) unable to explain observed nitrification rates (Fitzgerald et al., 2015). Thus, the recent discovery of comammox in a variety of environments (Daims et al., 2015; van Kessel et al., 2015) prompted us to investigate their presence in low DO BNR systems.

The gene encoding the alpha-subunit of ammonia monooxygenase (*amoA*) has been one of the most widely used markers for detection and quantification of AOB and AOA, as it facilitates functional analysis and reconstruction of phylogenetic relationships (Rotthauwe et al., 1997; Purkhold et al., 2000; Koops et al., 2006; Pester et al., 2012). The *amoA* of comammox is distinguishable from *amoA* sequences of AOB and AOA, and thus, it can be used to detect the presence of comammox in environmental samples (Daims et al., 2015; van Kessel et al., 2015; Pinto et al., 2016; Camejo et al., 2017). Recent descriptions of PCR primers targeting the *amoA* gene of commamox include a primer pair designed specifically for *Candidatus* (*Ca.*) Nitrospira inopinata (Daims et al., 2015), a primer pair designed specifically for a comammox-like clone within a freshwater aquaculture system (Bartelme et al., 2017), a primer collection that differentiates two broad clades of comammox within the *Nitrospira* genus (Pjevac et al., 2017), and a highly degenerate primer pair attempting to encompass all comammox within *Nitrospira* (Fowler et al., 2017). To date, three candidate comammox species have been described in the literature, namely *Ca.* N. nitrosa, *Ca.* N. inopinata, and *Ca.* N. nitrificans (Daims et al., 2015; van Kessel et al., 2015). However, quantifying the contribution of these candidate species to the comammox community in environmental samples has not been possible because of the lack of specific qPCR primer sets. To overcome this limitation, we designed a set of highly specific non-degenerate primers for the independent detection of *Ca.* N. nitrosa, *Ca.* N. inopinata, and *Ca.* N. nitrificans. We used these primers to evaluate the contribution of these species to the comammox population in samples from BNR plants operated at low DO conditions.

## Materials and Methods

### Sample Collection, Processing, and DNA Extraction

Environmental samples used in this study originated from four low DO nitrifying bioreactors: a laboratory-scale sequencing batch reactor (L_SBR), a pilot-scale sequencing batch reactor (P_SBR), a pilot scale continuous flow (P_CF) reactor simulating a University of Cape Town configuration without nitrate recycle, and a full-scale wastewater treatment plant (WWTP) from the Trinity River Authority (TRA) Central Region (Arlington, TX, United States) (Table 1). For comparison, samples were also collected from the full-scale Nine Springs WWTP (NS) at the Madison Metropolitan Sewerage District (Madison, WI, United States), which operates with typical high DO conditions (Table 1). Grab samples with varying volumes were collected from every reactor and were either centrifuged to form a biomass pellet, with supernatant discarded, and the pellet frozen at −80°C (P_SBR, P_CF, TRA, and NS) or were saved in a glycerol mixture prior to placement in a −80°C freezer (L_SBR). Biomass samples were stored at −80°C until DNA extraction. All DNA was extracted using DNeasy^®^ PowerSoil^®^ DNA Isolation Kit (Qiagen, Hilden, Germany) following the manufacturer’s directions. DNA was quantified with a Qubit fluorometer (Thermo Fisher Scientific, Waltham, MA, United States) and the purity ratio, or ratio of absorbance at 260 and 280 nm, was determined with a NanoDrop spectrophotometer (Thermo Fisher Scientific, Waltham, MA, United States). DNA samples were stored at −20°C until further processing.

**Table 1:** Bioreactor sample characteristics.

Bioreactor (Sample ID)	Location	Sample Dates^a^	Configuration at time of sampling	Size (gpd)	SRT (days)	Reference
Lab-scale sequencing batch reactor (L_SBR)	Madison, WI, United States	3/7/2013 (1), 3/18/2013 (2), 4/17/2013 (3), 10/3/2014 (4)	Sequencing batch reactor (SBR) operated with dissolved oxygen (DO) < 0.2 mg O_2_/L	0.5	80	Camejo et al., 2016
Pilot-scale sequencing batch reactor (P_SBR)	Madison, WI, United States	9/10/2015 (1), 11/10/2015 (2), 12/15/2015 (3), 4/25/2016 (4)	Sequencing batch reactor (SBR) operated with DO < 0.7 mg O_2_/L	130	80	Camejo et al., 2016
Continuous flow pilot plant (P_CF)	Madison, WI, United States	9/9/2015 (1), 11/18/2015 (2), 12/30/2015 (3), 4/26/2016 (4)	Modified University of Capetown (UCT) operated with DO < 0.5 mg O_2_/L	1200	10	Keene et al., 2017
Trinity River Authority Central Region Wastewater System Treatment Plant (TRA)^b^	Arlington, TX, United States	8/9/2016 (1), 7/28/2017^b^ (2), 7/28/2017 (3)	Anaerobic Aerobic (AO) operated with three zones. Zone 1: DO < 0.6 mg O_2_/L Zone 2: DO < 0.9 mg O_2_/L Zone 3: DO < 1.4 mg O_2_/L	123 M	10	This study
Madison Metropolitan Sewerage District Nine Springs Plant (NS)	Madison, WI, United States	9/9/2015 (1), 11/18/2015 (2), 12/30/2015 (3), 4/26/2016 (4)	Modified University of Capetown (UCT) operated with DO > 2.0 mg O_2_/L	40 M	10	Keene et al., 2017

*^a^Number in parentheses after each date is incorporated after the sample ID. ^b^TRA sample 2 and 3 were collected from different aeration basins.*

### Design of Primers to Detect Comammox Ammonia Monooxygenase Gene *amoA*

A collection of full-length *amoA* and full-length particulate methane monooxygenase (*pmoA*) gene sequences, obtained from the National Center for Biotechnology Information (NCBI) GenBank database (NCBI Resource Coordinators, 2017), were used for primer design. The full-length sequences included in the design are indicated by bold text in the phylogenetic tree in Figure 3, with the corresponding accession number in parenthesis. All *amoA* and *pmoA* sequences were aligned using the ‘AlignSeqs’ command in the DECIPHER “R” package (Wright, 2015, 2016). This aligned database was then submitted to DECIPHER’s Design Primers web tool (Wright et al., 2014). Sequences corresponding to *Ca.* N. nitrosa, *Ca.* N. inopinata, and *Ca.* N. nitrificans were individually selected as target groups for primer design, which used the following parameters: primer length ranging from 17 – 26 nucleotides with up to 1 permutation, PCR product amplicon length of 100 – 450 bp, 100% target group coverage, and without the Taq 3′-end Model option. The primer design tool also used the same reaction conditions in all cases: [Na^+^] 70 mM, [Mg^2+^] 3 mM, [dNTPs] 0.8 mM, annealing temperature (T_a_) of 64°C, [primers] 400 nM. Amplification products were verified by agarose (2%) gel electrophoresis with GelRed^TM^ Nucleic Acid Gel Stain (Biotium, Freemont, CA, United States).

### Quantitative Real-Time Polymerase Chain Reaction (qPCR)

Quantification of total 16S rRNA genes, total comammox *amoA* genes, clades A and B comammox *amoA*, as well as *Ca*. N. nitrosa, *Ca.* N. inopinata, and *Ca.* N. nitrificans *amoA* in each DNA sample was carried out by qPCR. All qPCR assays were performed on a Roche LightCycler^®^ 480 high-throughput real-time PCR system using white LightCycler^®^ 480 multiwell plates and the associated LightCycler^®^ 480 sealing foils (Roche Molecular Systems, Inc., Pleasanton, CA, United States). All environmental DNA samples were diluted to 10 ng/μL. Although not specifically studied here, the applied sample dilutions helped reduce any potential problems with PCR inhibition. All qPCR assays were prepared in Bio-Rad 2x iQ^TM^ SYBR^®^ Green Supermix (Bio-Rad, Hercules, CA, United States), containing 50 U/mL iTaq DNA polymerase, 1.6 mM dNTPs, 100 mM KCl, 40 mM Tris-HCl, 6 mM MgCl_2_, 20 nM fluorescein, and stabilizers (10 μL per reaction). Triplicate reactions were prepared for each sample.

Amplification of total comammox *amoA* was performed according to Fowler et al. (2017), with each reaction containing Bio-Rad 2x iQ^TM^ SYBR^®^ Green Supermix (10 μL), nuclease free water (4.4 μL), environmental DNA or standard (4 μL), and PCR primers (0.8 μL of 12.5 μM each primer) added for a final volume of 20 μL per reaction. The thermal cycling protocol for the total comammox primers was as follows: initial denaturation step at 94°C for 5 min, followed by 40 cycles of initial denaturation at 94°C for 30 s, annealing at 48°C for 30 s, and extension at 72°C for 1 min.

Amplification of clade A and clade B comammox *amoA* was performed using the equimolar primer mixtures according to Pjevac et al. (2017) (Table 2). Four equimolar primer mixtures were created for clade A and clade B forward and reverse primers, by combining 62.5 μL of each of the six individual primer stocks (100 mM) together with 125 μL nuclease free water to a final working volume of 500 μL and final concentration of 12.5 μM each primer. Finally, reactions were prepared in Bio-Rad 2x iQ^TM^ SYBR^®^ Green Supermix (10 μL), nuclease free water (4.4 μL), environmental DNA or standard (4 μL), and equimolar PCR primer mixture (0.8 μL of 12.5 μM each primer) were added to each reaction for a final volume of 20 μL per reaction. The thermal cycling protocol for the clade-level primers was as follows: initial denaturation step at 95°C for 3 min, followed by 45 cycles of initial denaturation at 95°C for 30 s, annealing at 52°C for 45 s, and extension at 72°C for 1 min.

**Table 2:** Primers used for qPCR.

Target gene	Primer name	Forward primer (5′ - 3′)	Reverse primer (5′ - 3′)	T_a_(°C)	Reference
*Ca.* Nitrospira nitrosa *amoA*	Nitrosa amoA-469F/812R	GCGATTCTGTTTTATCCCAGCAAC	CCGTGTGCTAACGTGGCG		
*Ca.* Nitrospira inopinata *amoA*	Inopinata amoA-410F/815R	TCACCTTGTTGCTAACTAGAAACTGG	TCCGCGTGAGCCAATGT	64	This study
*Ca.* Nitrospira nitrificans *amoA*	Nitrificans amoA-463F/836R	ATGTTCGCGGCACTGTT	CCAGAAAGTTTAGCTTTGTCGCCT		
Comammox *Nitrospira* clade A *amoA*	comaA-244f_a/659r_a	TACAACTGGGTGAACTA	AGATCATGGTGCTATG	52	Pjevac et al., 2017
	comaA-244f_b/659r_b	TATAACTGGGTGAACTA	AAATCATGGTGCTATG		
	comaA-244f_c/659r_c	TACAATTGGGTGAACTA	AGATCATGGTGCTGTG		
	comaA-244f_d/659r_d	TACAACTGGGTCAACTA	AAATCATGGTGCTGTG		
	comaA-244f_e/659r_e	TACAACTGGGTCAATTA	AGATCATCGTGCTGTG		
	comaA-244f_f/659r_f	TATAACTGGGTCAATTA	AAATCATCGTGCTGTG		
Comammox *Nitrospira* clade B *amoA*	comaB-244f_a/659r_a	TAYTTCTGGACGTTCTA	ARATCCAGACGGTGTG	52	Pjevac et al., 2017
	comaB-244f_b/659r_b	TAYTTCTGGACATTCTA	ARATCCAAACGGTGTG		
	comaB-244f_c/659r_c	TACTTCTGGACTTTCTA	ARATCCAGACAGTGTG		
	comaB-244f_d/659r_d	TAYTTCTGGACGTTTTA	ARATCCAAACAGTGTG		
	comaB-244f_e/659r_e	TAYTTCTGGACATTTTA	AGATCCAGACTGTGTG		
	comaB-244f_f/659r_f	TACTTCTGGACCTTCTA	AGATCCAAACAGTGTG		
Total comammox *Nitrospira amoA*	Ntsp-amoA 162F/359R	GGATTTCTGGNTSGATTGGA	WAGTTNGACCACCASTACCA	48	Fowler et al., 2017
Total *16S rRNA*	16S-341f/785r	CCTACGGGNGGCWGCAG	GACTACHVGGGTATCTAATCC	53	Klindworth et al., 2013; Thijs et al., 2017

For the novel *amoA* primers designed in this study (Table 2), nuclease free water (4.4 μL), environmental DNA or standard (4 μL), and PCR primers (0.8 μL of 10 μM each primer) were added to each reaction for a final volume of 20 μL per reaction. The thermal cycling protocol for qPCR using the novel *amoA* primers designed in this study (Table 2) was as follows: initial denaturation step at 95°C for 10 min, followed by 45 cycles of initial denaturation at 95°C for 10 s, annealing at 64°C for 30 s, and extension at 72°C for 30 s. Fluorescence was measured at 72°C for amplicon quantification. After amplification, an amplicon melting curve was recorded in 0.25°C steps between 65 and 97°C. Melting peaks were obtained by plotting the negative first derivative of fluorescence against temperature. Although 30 cycles is typically sufficient for quantification of targets in qPCR, the thermal cycling was extended to evaluate potential non-specific amplification with the newly designed primers (Wright et al., 2014). Finally, amplification of total 16S rRNA was performed using the 16S rRNA-targeted primer pair 341f/785r according to Thijs et al. (2017) (Table 2).

### Standard Preparation for qPCR

Full-length *amoA* from *Candidatus* Nitrospira nitrosa*, Ca.* N. inopinata, and *Ca.* N. nitrificans were used as standards for the new species-specific comammox assay as well as the total comammox and clade A comammox assays. Full-length *amoA* from *Nitrospira sp. CG24_E* (Palomo et al., 2018) was used as the standard for the clade B comammox assay. These individual *amoA* standards were generated from synthetic gene plasmid cloning vectors (Integrated DNA Technologies, Inc., Coralville, IA, United States) transformed into One Shot^TM^ TOP10 Competent *Escherichia coli* (Life Technologies, Carlsbad, CA, United States) with the TOPO-TA cloning kit (Invitrogen, Karlsruhe, Germany) which uses the pCR^TM^4-TOPO^®^ TA vector. The cloned plasmids were subjected to amplification with M13 primers and product sizes verified with gel electrophoresis. The M13-PCR products were purified using the Qiagen PCR Purification Kit (Qiagen, Hilden, Germany) and quantified using a Qubit fluorometer (Thermo Fisher Scientific, Waltham, MA, United States). The purity ratio of the standard was determined with a NanoDrop spectrophotometer (Thermo Fisher Scientific, Waltham, MA, United States).

Standard gene copy number was calculated from the DNA concentration. The standards were diluted in a 10-fold series over seven orders of magnitude (approximately 10^1^ to 10^7^ copies) and were included in triplicate with each set of samples. A 95% confidence interval and standard deviation was calculated for the fractional PCR cycle used for quantification (i.e., quantification cycle or C_q_) for each set of standard replicates. A regression line fit to the C_q_ and the known concentration of each standard was used to generate a standard calibration curve for each assay. Next, the standard replicate statistics and assay-specific standard calibration curve were used to approximate a limit of detection (LOD) and limit of quantification (LOQ) according to the Minimum Information for Publication of Quantitative Real-Time PCR Experiments (MIQE Guidelines) described by Bustin et al. (2009) and equations adapted from Armbruster and Pry (2008). Median LOD, median LOQ, and corresponding 95% confidence intervals are reported for each assay (Table 3). External standard curve amplification efficiencies were calculated as *E* = ($10^{\frac{-1}{m}}$ − 1) × 100, where m represents the slope of the standard curve.

**Table 3:** qPCR performance with three distinct comammox *amoA* standard templates.

	Primer set	Amplicon size (bp)	Amplicon GC content (%)	Average T_m_ (°C)	Log_10_ linear range, log(SQ)^∗^	Average PCR efficiency (%)	Median LOD^∗∗^ (copies/ng DNA)	Median LOQ^∗∗^ (copies/ng DNA)
Comammox	**Standard template: *N. nitrosa amoA***							
*Nitrospira* clade A	Nitrosa amoA-469F/812R^a^	344	57	88.8 ± 0.08	1 - 7	96 ± 3.0	*M* = 11, 95% CI [9.3, 13]	*M* = 13, 95% CI [10, 17]
	Ntsp-amoA 162F/369R^b^	198	54	86.1 ± 0.08	3 - 7	91 ± 2.6	*M* = 20, 95% CI [8.6, 48]	*M* = 28, 95% CI [11, 72]
	comaA-244f/659r^c^	415	58	88.9 ± 0.09	2 - 7	95 ± 2.7	*M* = 38, 95% CI [13, 110]	*M* = 40, 95% CI [14, 120]
	**Standard template: *N. inopinata amoA***							
	Inopinata amoA-410F/815R^a^	406	49	85.6 ± 0.05	1 - 7	90 ± 3.0	*M* = 7.0, 95% CI [4.0, 12]	*M* = 14, 95% CI [7.0, 29]
	Ntsp-amoA 162F/369R^b^	198	49	84.4 ± 0.60	3 - 7	82 ± 3.4	*M* = 20, 95% CI [8.6, 48]	*M* = 28, 95% CI [11, 72]
	comaA-244f/659r^c^	415	50	86.0 ± 0.10	3 - 7	86 ± 2.5	*M* = 38, 95% CI [13, 110]	*M* = 40, 95% CI [14, 120]
	**Standard template: *N. nitrificans amoA***							
	Nitrificans amoA-463F/836R^a^	374	52	86.7 ± 0.10	1 - 7	92 ± 2.4	*M* = 1.3, 95% CI [0.9, 2.0]	*M* = 3.8, 95% CI [1.3, 11]
	Ntsp-amoA 162F/369R^b^	198	57	87.3 ± 0.27	2 - 7	96 ± 1.8	*M* = 20, 95% CI [8.6, 48]	*M* = 28, 95% CI [11, 72]
	comaA-244f/659r^c^	415	56	88.2 ± 0.07	2 - 7	93 ± 2.9	*M* = 38, 95% CI [13, 110]	*M* = 40, 95% CI [14, 120]

*Average parameters are presented with standard deviation. PCR efficiency is reported for the linear range of the standard calibration curve. ^∗^SQ, starting quantity in copies per reaction. ^∗∗^M, median; CI, confidence interval [upper limit, lower limit]; ^a^This study. ^b^Fowler et al., 2017. ^c^Pjevac et al., 2017.*

### Gradient PCR

Gradient PCR was performed with the total comammox (Fowler et al., 2017) and clade-level comammox (Pjevac et al., 2017) *amoA* primers with environmental DNA samples diluted to three concentrations to determine if an optimal annealing temperature and DNA concentration exists to eliminate or at least minimize the observed non-specific amplification. Gradient PCR was performed on a Mastercycler^®^ nexus gradient PCR cycler (Eppendorf North America, Hauppauge, NY, United States) with Eppendorf twin.tec^®^ 96-well LoBind PCR plates and Eppendorf PCR sealing foils. Reaction mixtures and thermal cycling protocols were identical to the respective qPCR assay, with the exception that annealing temperatures were set to vary across each PCR plate. For the total comammox primers, annealing temperatures were set in small increments between 47.8 and 56°C. For the clade-level comammox primers, annealing temperatures were set in small increments between 51.8 and 60°C. Two samples were selected for each assay and were selected based on the presence of both the target and non-specific amplifications after qPCR. The total comammox gradient assay included DNA from P_SBR-4 and NS-4. The clade A and clade B comammox gradient assays included DNA from P_CF-4 and NS-4. All environmental DNA samples were diluted to 40 ng DNA/reaction, 20 ng DNA/reaction, and 1 ng DNA/reaction. Amplification products were then visualized with agarose (2%) gel electrophoresis with GelRed^TM^ Nucleic Acid Gel Stain (Biotium, Freemont, CA, United States).

### Phylogenetic Tree Construction

The database of aligned *amoA* and *pmoA* sequences was expanded by including additional comammox *amoA* sequences obtained from metagenomes available on the JGI Integrated Microbial Genomes (IMG) database (Markowitz et al., 2012) and available comammox *Nitrospira* bins extracted with mmgenome^1^ (Karst et al., 2016). The expanded database was utilized for *in silico* primer analyses and to construct a consensus phylogenetic tree in Geneious v. 9.1.2 (Kearse et al., 2012) using the Neighbor-Joining method on a Tamura-Nei genetic distance model with bootstrap resampling and 20,000 trials.

### Average Nucleotide Identity Calculation

To determine if comammox *Nitrospira* draft genomes belonged to the same species, we performed pairwise analyses of genome average nucleotide identity (ANI). Initially, open reading frames (ORFs) within each draft genome was predicted with Prodigal (Hyatt et al., 2010). Subsequently, the JSpecies Web Server (Richter et al., 2016) was used to calculate ANI based on BLAST+ (Camacho et al., 2009) from percent of aligned genome ORFs.

## Results

### Total Comammox and Clade-Level Comammox Detection

**FIGURE 1:**
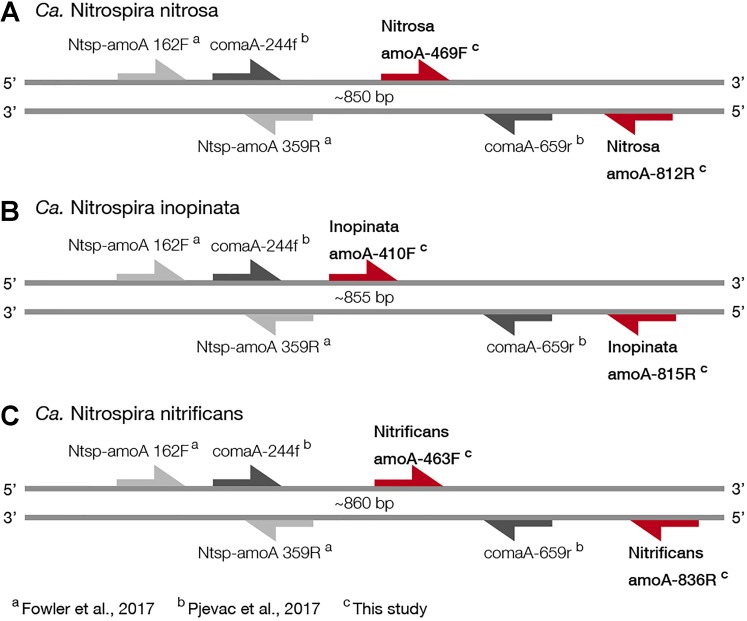
Visual representation of primer amplification regions with **(A)** *Ca.* N. nitrosa *amoA*, **(B)** *Ca.* N. inopinata *amoA*, and **(C)** *Ca.* N. nitrificans *amoA.* The new primers, labeled with red arrows, were designed using full-length *amoA* sequences and novel primer binding region was discovered near the end of each *amoA* gene (approximately between 400 and 800 bp). Base pair locations are indicated in the primer names. Most publicly available published *amoA* sequences are partial length and are located between approximately 244 and 659 bp. Amplification with clade A-specific equimolar primer mixtures occur in this region, while amplification with the total comammox primers occur between 162 and 359 bp.

For broad comammox *amoA* quantification, three published primer sets were compared using standards and nineteen environmental samples originating from five bioreactors (Table 1). The Ntsp-amoA 162F/359R primer set (Fowler et al., 2017), designed to target total comammox *Nitrospira amoA* (Table 2), amplifies a 198 bp fragment at primer binding regions between positions 162–182 and 339–359 bp (Figure 1). The comaA-244f/659R and comaB-244f/659r primer sets (Pjevac et al., 2017), designed to differentiate between clades A and B of comammox (Table 2), amplify a 415 bp fragment at *amoA* primer binding regions between positions 244–261 and 643–659 bp (Figure 1). The standard curves for these assays were linear over a minimum of five orders of magnitude and had high coefficients of determination (*R*^2^ > 0.990); although linear range, amplification efficiency, LOD and LOQ varied depending on the comammox *amoA* standard used (Table 3 and Supplementary Table S1). The results for the total comammox (Fowler et al., 2017) and clade-level comammox (Pjevac et al., 2017) assays are also summarized in decision matrices that were used to consistently evaluate qPCR results between assays (Supplementary Tables S3–S5).

**FIGURE 2:**
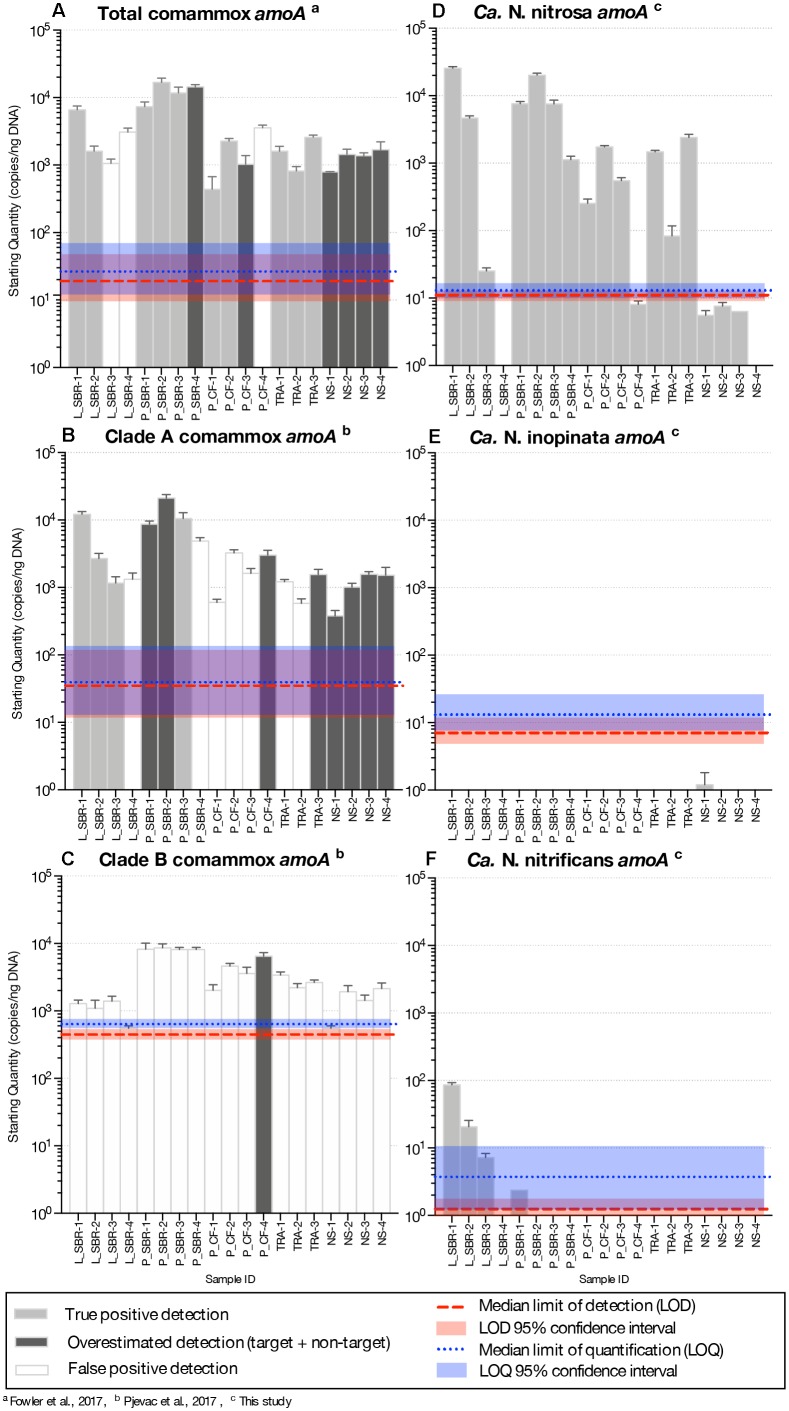
Abundance of *amoA* genes (gene copies/ng DNA) on a log-scale from comammox *Nitrospira* using the **(A)** total comammox primers, **(B)** clade A primers **(C)** clade B primers, **(D)** the newly designed *Ca.* N. nitrosa specific primers **(E)** *Ca.* N. inopinata specific primers and **(F)** *Ca.* N. nitrificans specific primers from various time series samples originating from five bioreactors. LS, lab-scale sequencing batch reactor; P_SBR, pilot-scale sequencing batch reactor; P_CF, continuous flow pilot plant; TRA, Trinity River WWTP; NS, Nine Springs WWTP. Error bars show the standard deviation of the triplicate samples. The color of each column indicates whether there was a true positive detection, an overestimated detection, or a false positive detection. The median limit of detection and quantification for each assay are represented by horizontal red and blue dotted lines, respectively. The shaded regions surrounding these limits represent the upper and lower bounds of the 95% confidence interval.

In the total comammox assay, melting curves for the standards were narrow and unimodal above ∼10^1^ – 10^2^ copies (Supplementary Figures S1A–C). However, melting curves from the environmental samples (Supplementary Figures S1D–H) and the agarose gel of qPCR products (Supplementary Figure S2) showed non-specific amplifications resulting in three false positive detections (amplification did not correspond to expected product size) and overestimation of comammox abundance (multiple products seen, including one with the expected product size) in six samples (Figure 2A and Supplementary Table S3).

In the clade A comammox assay, all standards with approximately 10^2^ *amoA* copies or less presented two melting peaks, the expected target peak at approximately 88.9, 86.0, and 88.2°C for *Ca.* N. nitrosa*, Ca.* N. inopinata, and *Ca.* N. nitrificans *amoA*, respectively, plus a sizeable off-target peak at approximately 70°C (Supplementary Figures S3A–C). Higher copy numbers produced narrow, unimodal melting peaks at the expected T_m_. The agarose gel performed with the qPCR products showed a single band for the standards; however, eight of the nineteen environmental samples contained multiple non-specific amplified products including a potential target amplicon (Supplementary Figures S3D–H, S4) leading to overestimated gene abundance (Figure 2B and Supplementary Table S4). Amplification of non-specific products in seven additional samples produced artifact-associated fluorescence that was well over the LOQ and translated into false positive predictions of the same order of magnitude as true positive detections (Figure 2B).

Clade B standards with approximately 10^3^ *amoA* copies or less also contained two melting peaks, an off-target peak at approximately 72°C and the expected target peak at approximately 89°C (Supplementary Figure S5A). In the clade B assay, fifteen samples had a target melting peak at the expected T_m_ (Supplementary Figures S5B–F), however, the agarose gel revealed that these samples had abundant non-specific products that were approximately 300 bp long— smaller than the expected 415 bp target size (Supplementary Figure S6). Only one sample (P_CF-4) appeared to contain an amplicon of the expected size (Supplementary Figure S6), but also contained several other non-specific amplicons. Thus, none of the samples tested were confirmed to be a true positive for presence of clade B comammox (Supplementary Table S5).

The biggest challenge with the non-specific amplification observed with the total comammox and clade-level comammox primers was the significant artifact-associated fluorescence resulting in C_q_ values within the same range as samples with abundant comammox (see Supplementary Tables S3–S5), which did not allow us to eliminate samples based on the LOD or LOQ. Two factors that could have contributed to the non-specific amplifications observed with the broad-detection primers are the low design annealing temperatures (48°C for the total comammox primers and 52°C for the clade A and clade B comammox primers) and the total DNA concentration we used in each assay. Therefore, we performed gradient PCR experiments to evaluate whether adjustments to the primer annealing temperature or sample DNA concentration would improve the specificity of the total comammox primers (Fowler et al., 2017) and the clade A and clade B comammox primer sets (Pjevac et al., 2017). In our specific application, the gradient PCR experiments show that small adjustments to annealing temperature or sample dilutions did not make a meaningful difference in the non-specific amplifications. Non-specific products remained in the samples tested at higher annealing temperatures and at all three DNA concentrations tested (40 ng/reaction, 20 ng/reaction, and 1 ng/reaction) with all three published primer sets. In the gradient PCR with the total comammox primers, non-specific products are observed at approximately 300 and 600 bp in the P_SBR-4 sample and at approximately 150, 350, 600, and 700 bp in the NS-4 sample, and do not appear to be dependent on the total DNA concentrations tested in the reaction (Supplementary Figure S7). In the gradient PCR for the clade A comammox primers, non-specific products are observed at approximately 150 and 300 bp in the P_CF-4 sample and at approximately 250 and 300 bp in the NS-4 sample, and the amplification of these non-specific products do not appear to be dependent on the total DNA concentrations tested in the reaction (Supplementary Figure S8). In the gradient PCR for the clade B comammox primers (Supplementary Figure S9), multiple non-specific products were observed, with the most prominent non-specific product consistent with previous observations at approximately 300 bp.

Another challenge we faced when applying the broad-detection comammox primers to our samples was the inability to rely on melting curve analysis alone to differentiate between true positive detections, false positive detections, and overestimated detections. To successfully complete melting curve analysis post qPCR, the melting characteristics of an amplified standard (characterized by the both the shape of the melting peak and the temperature where the peak occurs or T_m_) is compared to the melting peaks that appear in the environmental samples since the T_m_ considered a unique feature of a particular DNA fragment (Ririe et al., 1997; Wittwer and Kusakawa, 2004). However, this type of analysis can be difficult with primer sets that target a broad number of sequences, especially when the primers are designed to amplify a region with highly variable GC content. In this study, we found that the total comammox primers (Fowler et al., 2017) amplify a region of *Ca.* N. nitrosa, *Ca.* N. inopinata, and *Ca.* N. nitrificans *amoA* with GC content at 54, 49, and 57%, respectively. This produced melting peaks for *Ca.* N. nitrosa, *Ca.* N. inopinata, and *Ca.* N. nitrificans *amoA* at a T_m_ of 86.1°C, 84.4°C, and 87.3°C, respectively. We also found that the clade A comammox primers (Pjevac et al., 2017) amplify a region of *Ca.* N. nitrosa, *Ca.* N. inopinata, and *Ca.* N. nitrificans *amoA* with GC content at 58, 50, and 56%, respectively. This, in turn, produced melting peaks for *Ca.* N. nitrosa, *Ca.* N. inopinata, and *Ca.* N. nitrificans *amoA* at a T_m_ of 88.9, 86.0, and 88.2°C, respectively. While it is possible to determine a T_m_ range based on *in silico* analysis of the known comammox diversity, this does not allow us to eliminate non-specific products from the analysis since some may contain similar melting characteristics as the target. For example, the melting peaks for P_CF-4 with the total comammox primers (Supplementary Figure S1F) fall within the T_m_ range set by the standards used in this study, but only contained a non-specific product between 400 and 500 bp long when verified with agarose gel electrophoresis (Supplementary Figure S2). Since the T_m_ is also influenced by the reaction conditions, one would need to include a much greater number of standards *in situ* to represent a greater variety of known comammox species in order to truly rely on melting curve analysis alone with these broad-detection assays. Since more species are continually discovered, relying on melting curve analysis alone to confirm positive detections will likely be difficult without an array of comammox *amoA* standards and will require confirmation with an agarose gel electrophoresis of amplified qPCR products.

Overall, the presence, frequency, and influence of unspecific amplification in the total comammox (Fowler et al., 2017) assay and in the clade A and clade B (Pjevac et al., 2017) comammox assays were significant and did not allow for accurate detection and quantification of comammox *Nitrospira* in the specific environmental samples tested in this study (Figure 2A–C), prompting us to design other options for specific comammox detection.

### Design of Species-Specific Primer Sets

Since the broad range comammox primers did not provide satisfactory results with our environmental samples, we opted to focus our evaluations on the presence of specific comammox species. With this objective in mind, to detect and quantify comammox belonging to the *Candidatus Nitrospira* species currently described in the literature (*Ca.* N. nitrosa*, Ca.* N. inopinata, and *Ca.* N. nitrificans) we designed qPCR primer sets specifically targeting each of these species. For this design, we use a dataset of 27 full-length *amoA* gene sequences from AOB, AOA, and comammox, and 15 full-length *pmoA* gene sequences from methanotrophs. We limited the database to full-length *amoA* and *pmoA* sequences in order to allow for discovery of primer-binding regions outside of the fragments amplified by conventional *amoA* primer sets (Rotthauwe et al., 1997; Meinhardt et al., 2015). We used the Design Primers option in DECIPHER (Wright et al., 2014) with each one of the candidate species as the target group and other *amoA*/*pmoA* sequences entered as closely related groups that should not be amplified. In addition, for all designs, the annealing temperature and PCR conditions were fixed (see Materials and Methods) so that all three species-level primer sets could be simultaneously used in a single thermocycler run. For these species-specific primers, we take advantage of a higher annealing temperature (T_a_ = 64°C) than what was used for the total comammox (T_a_ = 48°C) and clade-level comammox (T_a_ = 52 °C) primers, which is a strategy that also helps improve primer specificity.

For *Ca.* N. nitrosa, the two *amoA* copies identified in the *Ca.* N. nitrosa genome (van Kessel et al., 2015) were used as the target group. The design algorithm identified primer binding regions at positions 469–493 and 794–812, predicting amplification of a 344 bp fragment (Figure 1). The target regions for each primer had 5 or greater mismatches to all other sequences in the design database, predicting a 100% specificity to the target group.

A single sequence (Daims et al., 2015) served as the target group for the design of *Ca.* N. inopinata primers, resulting in primer binding sites at 410–436 and 798–815, for a 406 bp fragment amplification. The target region for each primer had 5 or greater mismatches to other *amoA* and *pmoA* sequences used as non-targets in the design database, also predicting 100% specificity.

For *Ca.* N. nitrificans, two sequences were used as targets; the one in the *Ca.* N. nitrificans genome (van Kessel et al., 2015) and the one from *Nitrospira* sp. Ga0074138 (Pinto et al., 2016), which had 91.4% sequence identity to the *Ca.* N. nitrificans sequence. The primers amplified a 374 bp fragment, between positions 463 and 836. The predicted specificity was again 100%, with 5 or greater mismatches per primer to other sequences used in the design database.

**FIGURE 3:**
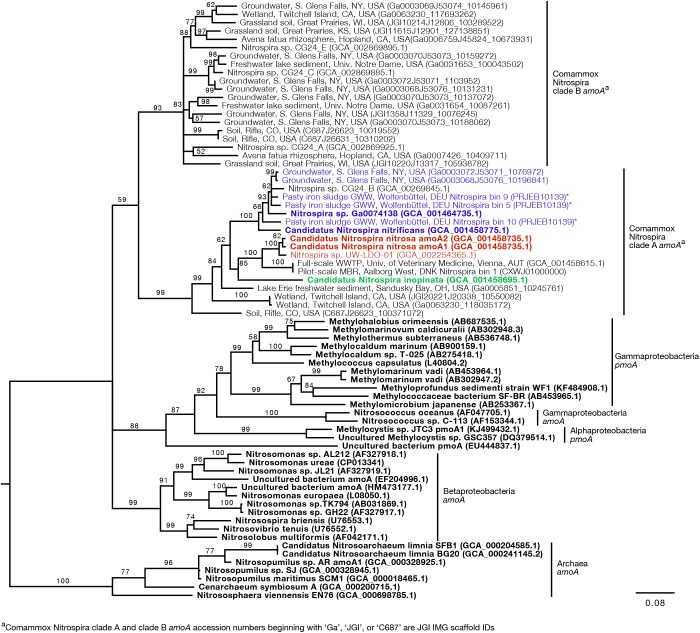
Neighbor-joining consensus tree generated from an alignment of full-length and near full-length *amoA* and *pmoA* gene sequences (>600 bp), rooting with archaea *amoA*. Bootstrap values, shown at the nodes where the value was greater than 50, are based on 10,000 trials. Bold sequence names were included in the new primer design. Blue, red, and green text are amplified by the *Ca.* N. nitrificans, *Ca.* N. nitrosa, and *Ca.* N. inopinata primer sets, respectively. Asterisks indicate that the gene is amplified but with a reduced efficiency due to base pair mismatches. The scale bar indicates the number of nucleotide substitutions per site. Accession numbers are presented after the sequence names. Acronyms were used for groundwater well (GWW), wastewater treatment plant (WWTP), and membrane bioreactor (MBR).

Since the sequence dataset used for design was small, we searched for additional *amoA* sequences that would contain the target regions of the new primer sets. However, this analysis was limited because most of the published *amoA* sequences contain the target side of the forward primers, but not the site targeted by the reverse primer (Figure 1). In this search, we found 33 nearly full-length sequences that not only clustered with the comammox sequences but were also long enough to contain the target regions of the species-specific primers (Figure 3). This set of nearly full-length *amoA* sequences included *amoA* sequences from comammox metagenomes that have been recently described in the literature (Daims et al., 2015; van Kessel et al., 2015; Palomo et al., 2016; Pinto et al., 2016; Camejo et al., 2017; Pjevac et al., 2017).

**FIGURE 4:**
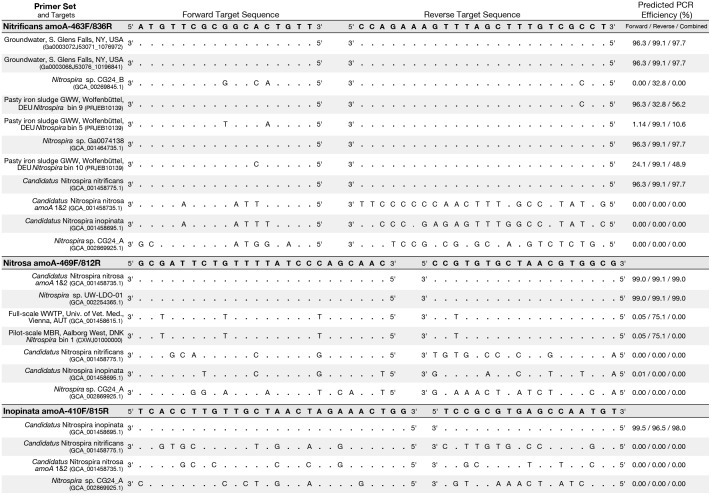
Primer-target mismatch analysis for the newly designed primers and near full-length comammox clade A *amoA* greater than 600 bp. Predicted PCR efficiency is reported separately for the forward and reverse primers in addition to a combined amplification efficiency. Dots indicate a base match and letters indicate a mismatch, with the letter indicating which base is actually present in the sequence.

**FIGURE 5:**
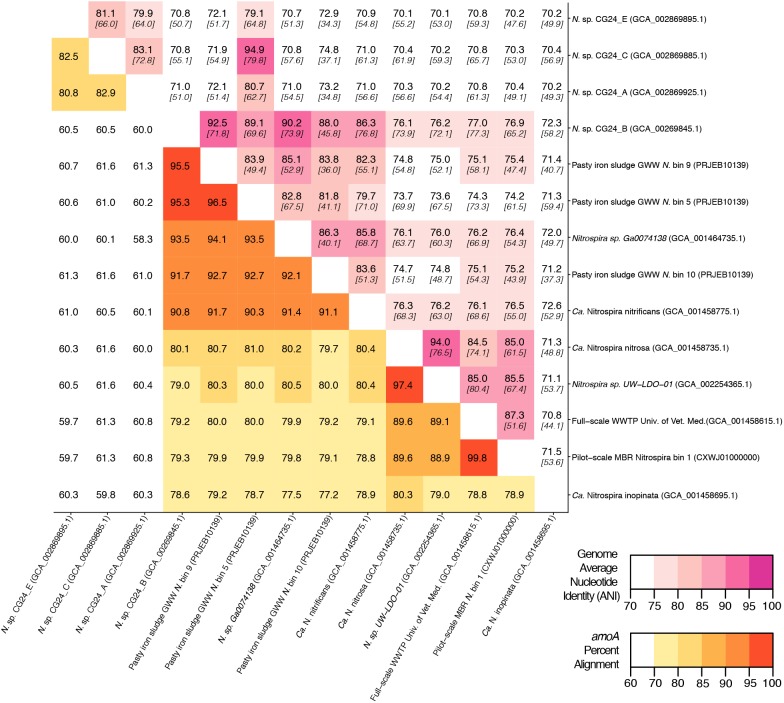
Matrix demonstrating the percent alignment of *amoA* genes (white to yellow to red gradient located on the bottom left portion of the matrix) and average nucleotide identity (ANI) of whole genome open reading frame alignments (white to pink gradient located on the top right portion of the matrix). Here, the percent genome ANI is shown in bold on the top of each square and fraction aligned is shown italicized and in brackets beneath the ANI value.

An *in silico* primer-target analysis (Figure 4) shows that *N. sp.* UW-LDO-01 (Camejo et al., 2017), originally described as a strain of *Ca.* N. nitrosa, has perfect matches to the newly designed primers targeting this species. Two *amoA* sequences (Full scale WWTP, Univ. of Veterinary Medicine, Vienna, AUT, GCA_001458615.1; Pilot-Scale MBR, Aalborg West, DNK Nitrospira bin 1, CXWJ01000000) clustered together and near the *Ca.* N. nitrosa cluster (Figure 3). The ANI of their genomes compared to *Ca.* N. nitrosa is 84.5 and 85%, respectively (Figure 5), lower than the typical cutoff for species definition (ANI > 94%) (Richter and Rossello-Mora, 2009). In agreement, we predict that their *amoA* will not be amplified by the nitrosa-specific primer set due to multiple mismatches with forward and reverse primers (Figure 4). The *amoA* sequence of *Nitrospira* sp. CG24_B (Palomo et al., 2016) clustered with *Ca.* N. nitrificans (Figure 3). However, the ANI of these two genomes (86.3%; Figure 5) is below the typical cutoff for species definition and the *in silico* analysis predicts that *Nitrospira* sp. CG24_B *amoA* will not be amplified with the nitrificans-specific primers (Figure 4). Three additional *amoA* sequences clustering with *Ca.* N. nitrificans include those from the draft genomes of ‘Pasty iron sludge GWW N. bin 5, N. bin 9, and N. bin 10 (PRJEB10139)’ (Daims et al., 2015) (Figure 3), which contain fewer mismatches (Figure 4) and are predicted to partially amplify (below 56% efficiency) with the *Ca.* N. nitrificans primers despite ANI below the species cutoff (Figure 5). Overall, the *amoA* phylogeny close to *Ca.* N. nitrificans remains unresolved, and therefore, the designed primer set for this species will likely require future refinement.

Sequences from clade B comammox were not included in dataset used for primer design, and therefore, an *in silico* analysis was also performed with these sequences (Figure 4 shows *N. sp*. CG24_A *amoA* as an example of clade B comammox). In all cases, the sequences had greater than 4 mismatches per primer and are predicted to not amplify with any of the new species-specific primer sets.

The standard curves for all three primer sets were linear (correlation coefficient *R*^2^ ≥ 0.996) over 7 orders of magnitude (Supplementary Table S1). The amplification efficiency for *Ca.* N. nitrosa, *Ca.* N. inopinata, and *Ca.* N. nitrificans *amoA* was 96 ± 3.0%, 90 ± 3.0%, and 92 ± 2.4%, respectively (Table 3). Melting curve analysis of all standards showed amplification of the target product without primer dimer artifacts, represented by a strong fluorescence signal producing a single melting peak at approximately 88.8°C for *Ca.* N. nitrosa (Supplementary Figures S10A, S11A), 85.6°C for *Ca.* N. inopinata (Supplementary Figures S12A, S13A), and 86.7°C for *Ca.* N. nitrificans (Supplementary Figures S14A, S15A).

### Environmental Detection of Candidate Comammox Species

Using the new species-specific primer sets developed in this study (Nitrosa amoA-469f/812r, Inopinata amoA-410f/815r, and Nitrificans amoA463f/836r), we evaluated the samples originating from the low DO BNR bioreactors (Table 1). With the new primers, positive detections obtained with melting curve analysis (Supplementary Figures S10–S15) correlated well with a positive target amplicon in the agarose gel (Supplementary Figures S16–S18). Additionally, cross-hybridization and primer dimers were not observed following PCR with agarose gel electrophoresis with the standard templates (Supplementary Figure S19). The results from the new species-specific primers are also summarized in decision matrices that were used to consistently evaluate qPCR results between assays (Supplementary Tables S6–S8).

Comammox *amoA* belonging to *Ca.* N. nitrosa were detected in all environmental samples. Agarose gel electrophoresis of the qPCR amplified products validated these results, since a single amplicon with the expected length (344-bp) was obtained from all 19 samples (Supplementary Figure S17). *Ca.* N. nitrosa abundance was greater than 10^3^ copies *amoA*/ng DNA in nine samples originating from low DO bioreactors L_SBR, P_SBR, P_CF, and TRA (Figure 2D). The maximum number of *amoA* copies was obtained from L_SBR-1, with approximately 2.5 × 10^4^ copies *amoA*/ng DNA (Figure 2D). After 45 cycles, six samples (L_SBR-4, P_CF-4, NS-1, NS-2, NS-3, and NS-4) were estimated to have fewer copies of *amoA* per ng DNA than the calculated LOD (*M* = 11, 95% CI [9.3, 13] copies/ng DNA) (Figure 2D and Table 3). However, these positive qPCR detections were confirmed via agarose gel electrophoresis (Supplementary Figures S16A, S17), suggesting that the *Ca.* N. nitrosa primers are highly sensitive (Supplementary Table S6). The melting curve analysis at 45 cycles (Supplementary Figure S10) and 30 cycles (Supplementary Figure S11) also confirmed the positive qPCR detections.

Compared to the quantification of total comammox (Fowler et al., 2017), *Ca.* N. nitrosa comprised an average of 340 ± 70%, 96 ± 29%, 67 ± 13%, and 93% of the total comammox population in the true positive detections obtained from L_SBR, P_SBR, P_CF, and TRA, respectively (Figure 2A,D). Although in some cases the lower quantification with the new species-specific primers could indicate the presence of other non-targeted comammox species, we also see that the total comammox primers contain 4 total mismatches to the primer-target region of *Ca.* N. nitrosa, since mismatches were allowed in the original total comammox primer design (Supplementary Table S2). In addition, we observed reduced total comammox (Fowler et al., 2017) efficiency when *Ca.* N. nitrosa *amoA* was present at less than 10^3^ copies (Supplementary Table S1). Thus, total comammox (Fowler et al., 2017) primer mismatches to *Ca.* N. nitrosa *amoA* may contribute to an overall reduced amplification efficiency and underestimation of this species in environmental samples that contain an abundant comammox population, like L_SBR and P_SBR. All in all, the new species-specific primers show that *Ca.* N. nitrosa is an important member of the comammox population in wastewater treatment plants and may contribute to low oxygen nitrification (as seen in L_SBR, P_SBR, P_CF and TRA).

Comammox *amoA* belonging to *Ca.* N. inopinata and *Ca.* N. nitrificans were less frequent and were only detected with less than 10^2^ copies *amoA*/ng DNA in all samples (Figure 2E,F and Supplementary Figure S18). N. inopinata *amoA* was positively detected in one sample from the Madison Metropolitan Sewerage District Nine Springs plant (NS), NS-1 (Supplementary Table S7); although, the concentration of N. inopinata *amoA* was less than the calculated LOD (Figure 2E and Table 3). *Ca.* N. nitrificans was only detected above the LOQ in L_SBR-1 and L_SBR-2 with approximately 86 and 21 *amoA*/ng DNA, respectively (Figure 2F and Supplementary Table S8). Overall, comammox *amoA* belonging to *Ca.* N. inopinata or *Ca.* N. nitrificans were minor contributors to the total bacteria population after normalization (with relative abundances less than 0.03%) (Supplementary Figure S20).

With the new species-specific primers, we intentionally ran amplifications for 45 cycles, to make sure we could detect any potential non-target amplification. Consequently, the melting curve analysis show some peaks in samples that did not have the target (Supplementary Figures S12B–F, S14C–F), but these peaks corresponds to noise after a large number of cycles that would not be seen in quantifications with a more conventional number of cycles. To verify that the noise in the melting peaks was due to the large number of cycles, we repeated the qPCR experiment with just 30 cycles, which eliminated the peaks that corresponded to noise (Supplementary Figures S11, S13, S15). Agarose gel electrophoresis of the qPCR amplified products after 30 cycles reaffirm the specificity of the new species-specific primers (Supplementary Figure S16). Moreover, samples that contained noise in the melting peaks when the qPCR was performed with 45 cycles occurred at a late C_q_, and consequently, any artifact associated fluorescence was minimal. If these samples had been quantified, the concentrations would be below both the LOD and LOQ, making them easy to eliminate from further analysis without needing to confirm with agarose gel electrophoresis (Supplementary Tables S6–S8).

## Discussion

Since the late 1800s, nitrification had been described as a division of labor between two phylogenetically distinct and specialized chemolithoautotrophs, the ammonia oxidizers and the nitrite oxidizers (Brock, 1999). This perception was not challenged for over a century, until Costa et al. (2006) hypothesized that a single nitrifying bacterium combining ammonia oxidation and nitrite oxidation should exist in nature. Nearly one decade later, in late 2015, two separate research groups discovered, cultivated, and characterized the ‘missing’ comammox organism from an aquaculture system and a deep oil exploration well, respectively (Daims et al., 2015; van Kessel et al., 2015). To date, there is still uncertainty about the occurrence of the novel *Nitrospira-*like comammox organisms in full scale activated sludge systems. Some studies suggest that comammox are not relevant to conventional wastewater treatment (Chao et al., 2016; Gonzalez-Martinez et al., 2016). However, since comammox were discovered in low oxygen environments (Daims et al., 2015; van Kessel et al., 2015), they may be able to efficiently utilize oxygen and may be important to low DO BNR, as recently described by Camejo et al. (2017).

Long-term low DO total nitrogen removal has been successfully demonstrated in laboratory (Camejo et al., 2016) and pilot-scale (Camejo et al., 2016; Keene et al., 2017) bioreactors seeded with Nine Springs WWTP activated sludge and operated with DO concentrations less than 0.60 mg O_2_/L. Samples analyzed from the Nine Springs WWTP showed presence of *Ca.* N. nitrosa, but below the LOQ. The sludge from this WWTP was the seed for the L_SBR, P_SBR, and P_CF reactors, all of which showed presence of *Ca.* N. nitrosa, suggesting that the low DO conditions favored accumulation of *Ca.* N. nitrosa as a potential participant in ammonia oxidation in low DO BNR. Interestingly, *Ca.* N. nitrosa was also abundant in the full-scale TRA system, which is operated with low DO conditions (Table 1), providing independent support for the hypothesis that *Ca.* N. nitrosa is an important contributor to ammonia oxidation in WWTP operated with low DO. *Ca.* N. nitrificans was detected above the LOQ in the L_SBR reactor only, whereas *Ca.* N. inopinata was not detected above the LOD in any of the low DO reactors; therefore, out of the three candidate species, *Ca.* N. nitrosa appears to be the only one that becomes enriched under low DO conditions.

When compared to quantification of the total bacterial population by 16S rRNA-targeted qPCR, the greatest relative abundance of *Ca.* N. nitrosa was 10% and 6% of total bacteria in samples originating from the laboratory-scale and pilot-scale sequencing batch reactors, respectively (Supplementary Figure S20). Although these two reactors were seeded with the same sludge, they were fed from completely different sources (synthetic media for L_SBR and full-scale primary effluent for P_SBR), but operated with low oxygen and a long solids retention time (SRT, both 80 days) (Camejo et al., 2016). The continuous flow reactors (P_CF and TRA) had lower relative abundances of *Ca.* N. nitrosa, and although operated with low DO, they had a much shorter SRT (both 10 days; Table 1). Thus, in addition to oxygen, SRT may be a factor that contributes to comammox abundance in low DO reactors.

Detection and quantification of microorganisms via real-time PCR relies on designing primers with good specificity to the targeted organisms, good coverage of the targeted group, and good quality of the experimental results. With any newly discovered target group, the quality of primer sets to achieve accurate quantification depends on the quality of the databases used for design and the design considerations. As more sequences of the targeted organisms become available, designs can only improve. The first primer design for comammox quantification described sets for targeting clade A and B *amoA* within the *Nitrospira* genus (Pjevac et al., 2017), and subsequently another set of primers was published, aiming for greater coverage to target all comammox *amoA* (Fowler et al., 2017). Our evaluation of these primer sets in environmental samples from low DO BNR plants showed challenges with primer dimer formation when target sequences were not abundant (clade A and clade B primers, Pjevac et al., 2017), unspecific amplifications in environmental samples (all sets), and underestimation with some strains due to primer-target mismatches (total comammox, Fowler et al., 2017). These challenges are more important when designing for broad target groups. In general, melting curve analyses of qPCR results are helpful for detection of nonspecific PCR products since different fragments will typically appear as distinct melting peaks (Ririe et al., 1997; Wittwer and Kusakawa, 2004). However, the broad detection and quantification of comammox *amoA* has high GC content variability among the comammox organisms described thus far (Table 3). Therefore, a wide range of amplicon melting temperatures are expected when using the total comammox (Fowler et al., 2017) or clade-level (Pjevac et al., 2017) assays, making melting curve analyses difficult to interpret. We found that verifying amplified products with the total comammox (Fowler et al., 2017) or clade-level (Pjevac et al., 2017) primers cannot be completed with melting curve analysis alone and will require a subsequent agarose gel electrophoresis of our environmental samples, which is not ideal for routine qPCR applications.

Setting the challenge of broad comammox quantification aside, we aimed at designing primer sets specific to the three candidate species described thus far. Evidently, there is a very small number of sequences representative of these candidate species; therefore, the designed primers inherently have 100% coverage. Specificity depends on finding sufficient differences between target and non-targets sequences (Wright et al., 2014), and the results showed this was possible for the three candidate species targeted. As more sequences become available from metagenome assemblies, the specificity of these primers can be re-evaluated. The beginning of such activity was performed in this study (Figure 4). Importantly, the qPCR experiments with the newly designed primers were conducted with a longer number of thermal cycles than typical (45 cycles) to increase the chances of detecting potential non-specific amplifications. This extended thermal cycling confirmed that that the primers are highly specific and are not producing unwanted non-specific amplifications.

Taken together, the species-specific comammox primers designed in this study enabled an analysis of comammox abundance focused solely on the candidate comammox species currently described in the literature. Although a narrowly focused analysis by design, it eliminated the problems of unspecific amplifications that compromised the use of broad comammox primers and resulted in strong experimental evidence in support of *Ca.* N. nitrosa being a comammox organism in energy-efficient BNR systems operated under low DO conditions. Given earlier studies on low-DO BNR providing some evidence of AOB also present in these systems (Fitzgerald et al., 2015; Keene et al., 2017) and no evidence of AOA (Fitzgerald et al., 2015), we hypothesize that *Ca.* N. nitrosa has an important contribution to ammonia oxidation in low-DO BNR reactors.

## Data Availability Statement

All datasets analyzed in this study are included in the manuscript and the Supplementary Files.

## Author Contributions

NB and DN developed the research plan and project goals. NB designed the primers and performed the laboratory work. Both NB and DN drafted the manuscript, tables, and figures. DN contributed sources of project funding. All authors have critically read, corrected, and approved the final version of the manuscript and agreed with the opinions expressed here.

## Conflict of Interest Statement

The authors declare that the research was conducted in the absence of any commercial or financial relationships that could be construed as a potential conflict of interest.

## Footnotes

1 http://madsalbertsen.github.io/mmgenome/
