# Supplementary material for: Design and Assessment of Species-Level qPCR Primers Targeting Comammox
Source: Front Microbiol. 2019 Jan 31;10:36. doi: 10.3389/fmicb.2019.00036 (PMC6365651; doi:10.3389/fmicb.2019.00036)
Supplement: Supplementary file 1 [file Data_Sheet_1.pdf]

Supplementary Material

Design and assessment of species-level qPCR primers targeting comammox

Natalie K. Beach and Daniel R. Noguera *

Department of Civil and Environmental Engineering, University of Wisconsin-Madison

*** Correspondence:**
Daniel R. Noguera, 1415 Engineering Drive, Madison, WI 53706
E-mail: noguera@engr.wisc.edu; Tel: 608-263-7783; Fax: 608-262-5199

1 Supplementary Figures and Tables

1.1 Supplementary Figures

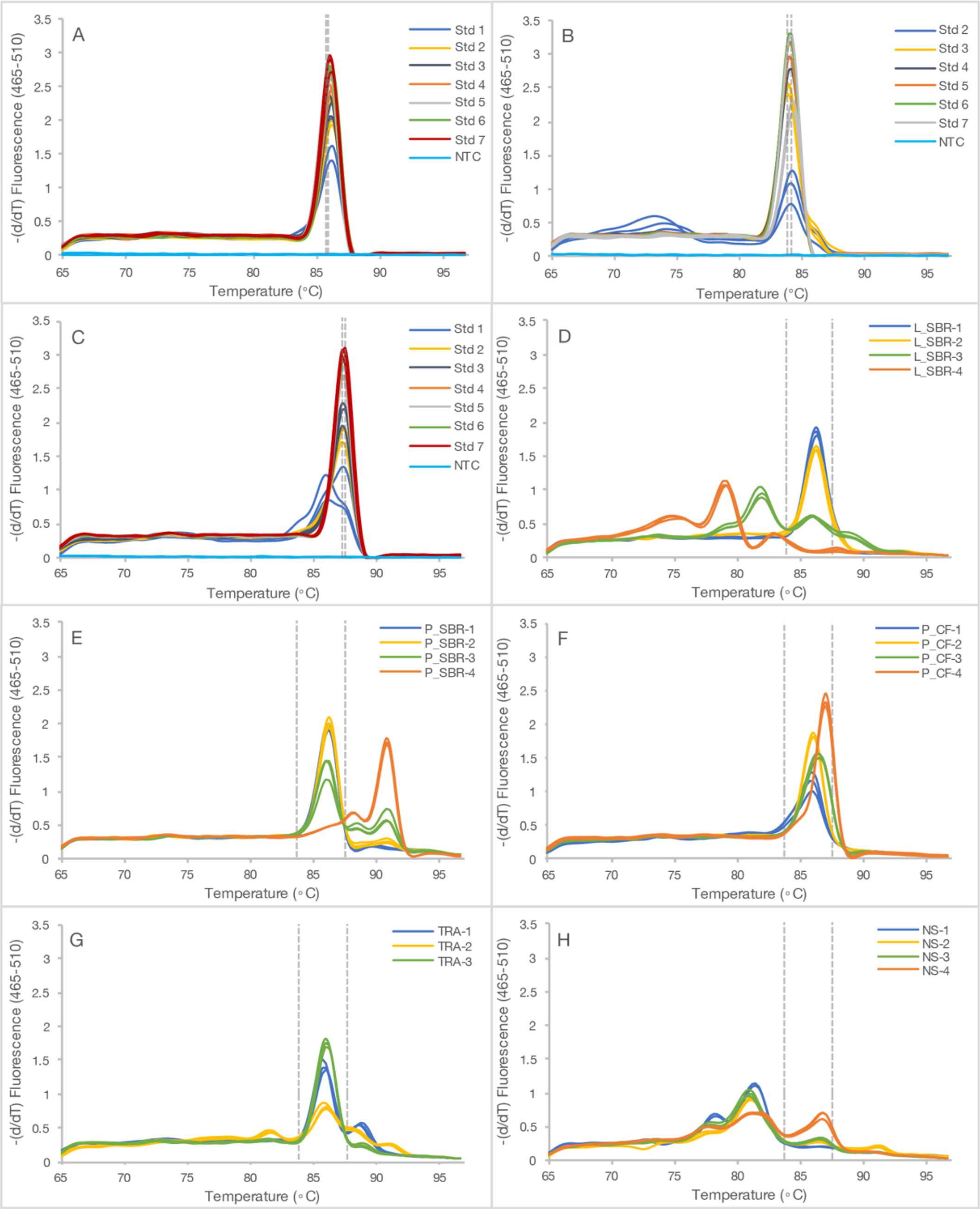

Figure S1. Total comammox assay (Ntsp-amoA 162F/359R) melting peaks for A) *Ca.* N. nitrosa *amoA* standard curve, B) *Ca.* N. inopinata *amoA* standard curve, and C) *Ca.* N. nitrificans *amoA* standard curve, D) L-SBR samples, E) P-SBR samples, F) P-CF samples, G) TRA samples, and H) NS samples. The dashed lines in panels A-C represent the standard deviation of melting temperatures for that specific standard. The dashed lines in panels D-H represent the minimum and maximum range of melting temperatures derived from all the standard curves. Triplicate data series are shown for each sample.

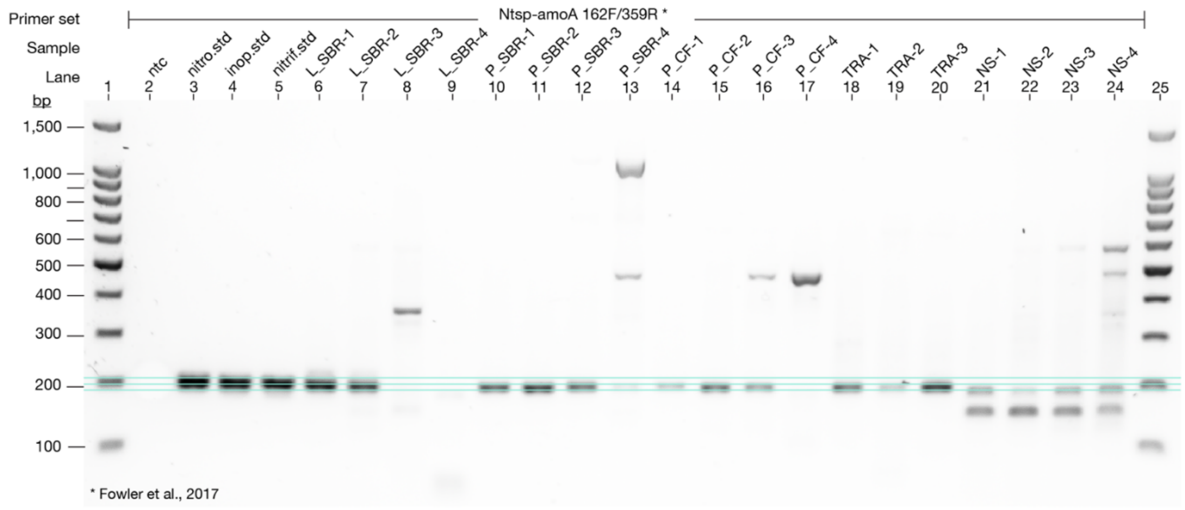

Figure S2. Agarose gel electrophoresis was performed using the total comammox (Ntsp-amoA 162F/359R) assay and included one replicate from each of the following samples: a non-template control (ntc), *Ca.* N. nitrosa *amoA* standard (nitro.std), *Ca.* N. inopinata *amoA* standard (inop.std), *Ca.* N. nitrificans *amoA* standard (nitrif.std), and one replicate from each of the 19 bioreactor samples. The expected amplicon size is 198 bp for this assay.

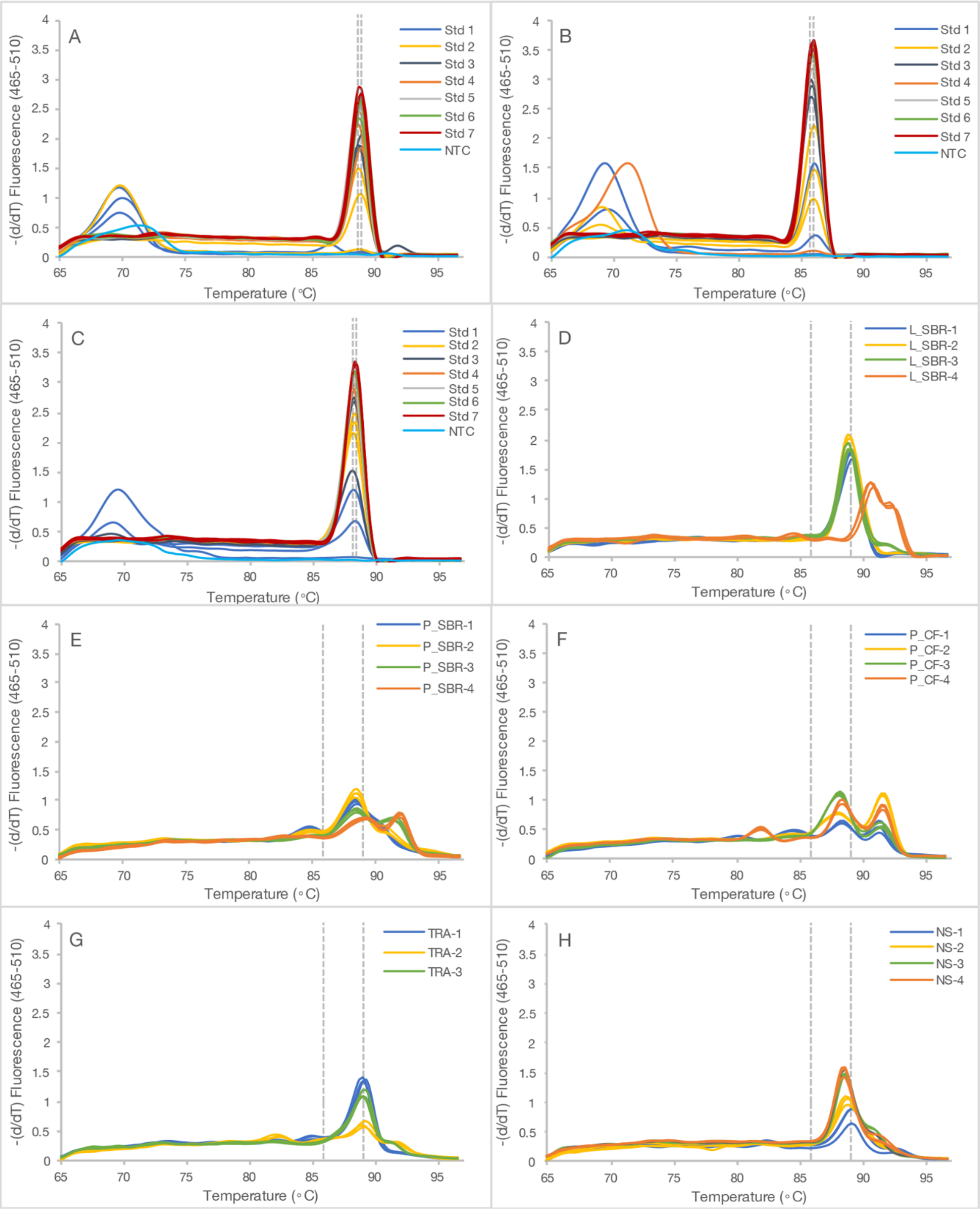

Figure S3. Clade A comammox assay (coma-244f/659r) melting peaks for A) *Ca.* N. nitrosa *amoA* standard curve*,* B) *Ca.* N. inopinata *amoA* standard curve*,* C) *Ca.* N. nitrificans *amoA* standard curve, D) L-SBR samples, E) P-SBR samples, F) P-CF samples, G) TRA samples, and H) NS samples. The dashed lines in panels A-C represent the standard deviation of melting temperatures for that specific standard. The dashed lines in panels D-H represent the minimum and maximum range of melting temperatures derived from all the standard curves. Triplicate data series are shown for each sample.

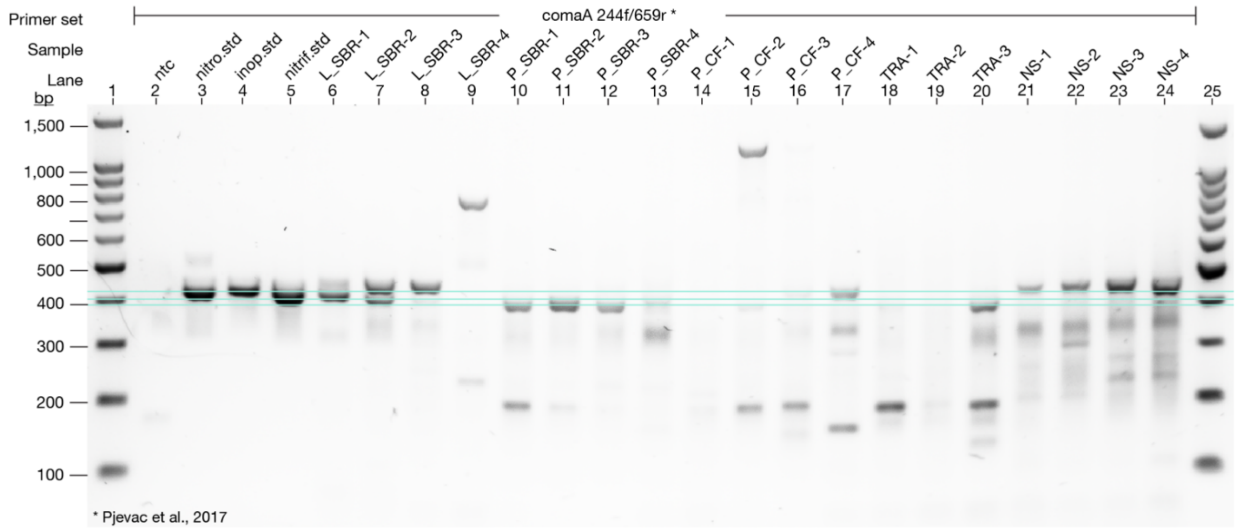

Figure S4. Agarose gel electrophoresis was performed using the clade A comammox (comaA-244f/659r) assay and included one replicate from each of the following samples: a non-template control (ntc), *Ca.* N. nitrosa *amoA* standard (nitro.std), *Ca.* N. inopinata *amoA* standard (inop.std), *Ca.* N. nitrificans *amoA* standard (nitrif.std), and one replicate from each of the 19 bioreactor samples. The expected amplicon size for this assay is 415 bp.

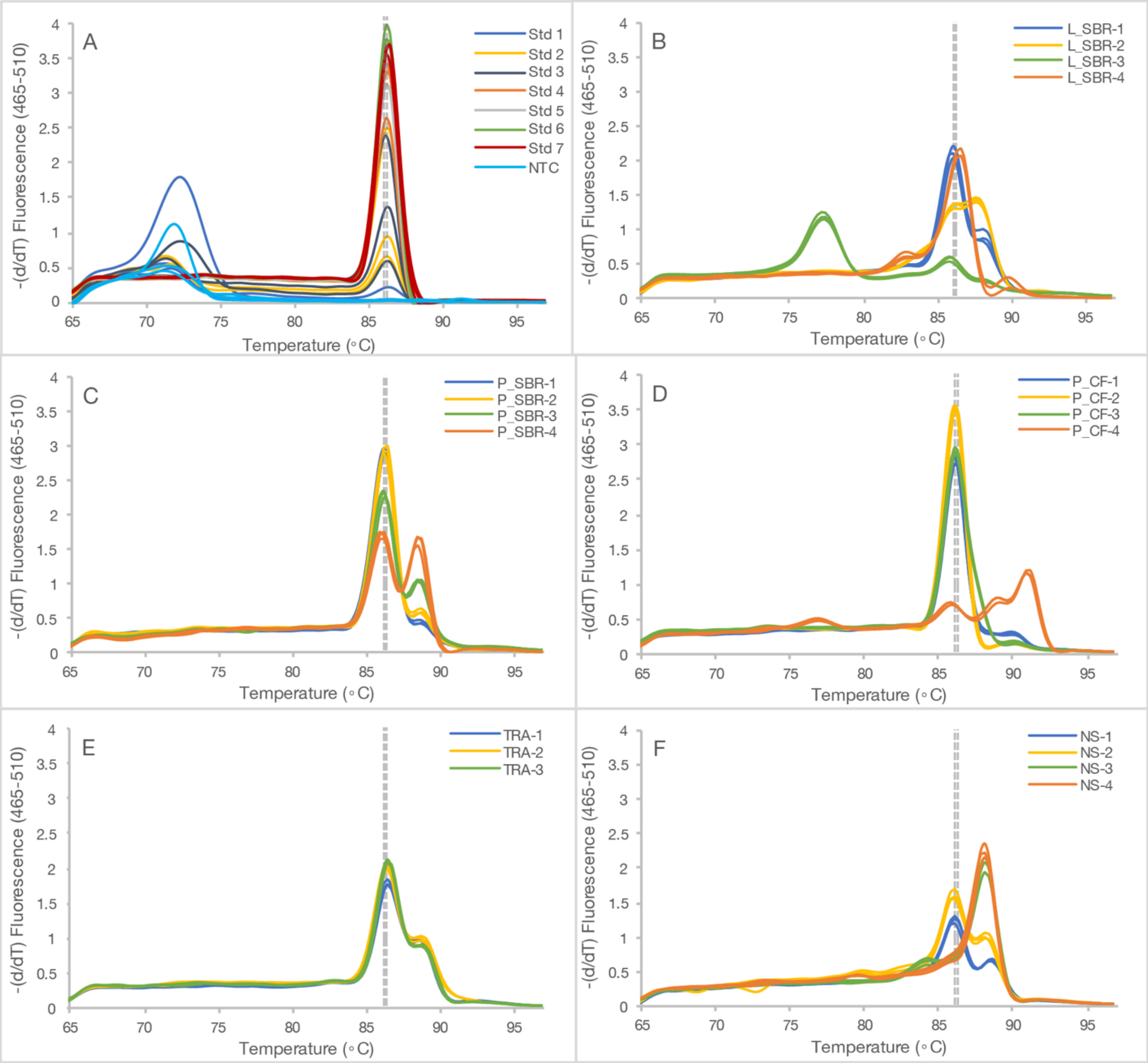

Figure S5. Clade B comammox assay (coma-244f/659r) melting peaks for A) *Nitrospira* sp CG24_E *amoA* standard curve*,* B) L-SBR samples, C) P-SBR samples, D) P-CF samples, E) TRA samples, and F) NS samples. The dashed lines in all panels represent the standard deviation of melting temperatures for the standard. Triplicate data series are shown for each sample.

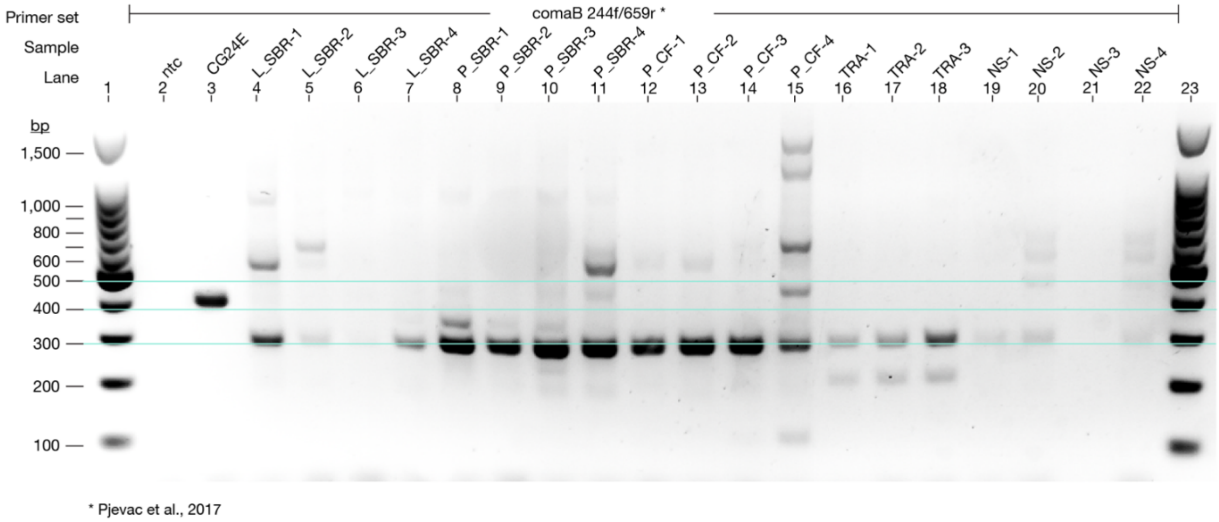

Figure S6. Agarose gel electrophoresis was performed using the clade B comammox (comaB-244f/659r) assay and included one replicate from each of the following samples: a non-template control (ntc), *Nitrospira* sp. CG24_E *amoA* standard (CG24E), and one replicate from each of the 19 bioreactor samples. The expected amplicon size for this assay is 415 bp

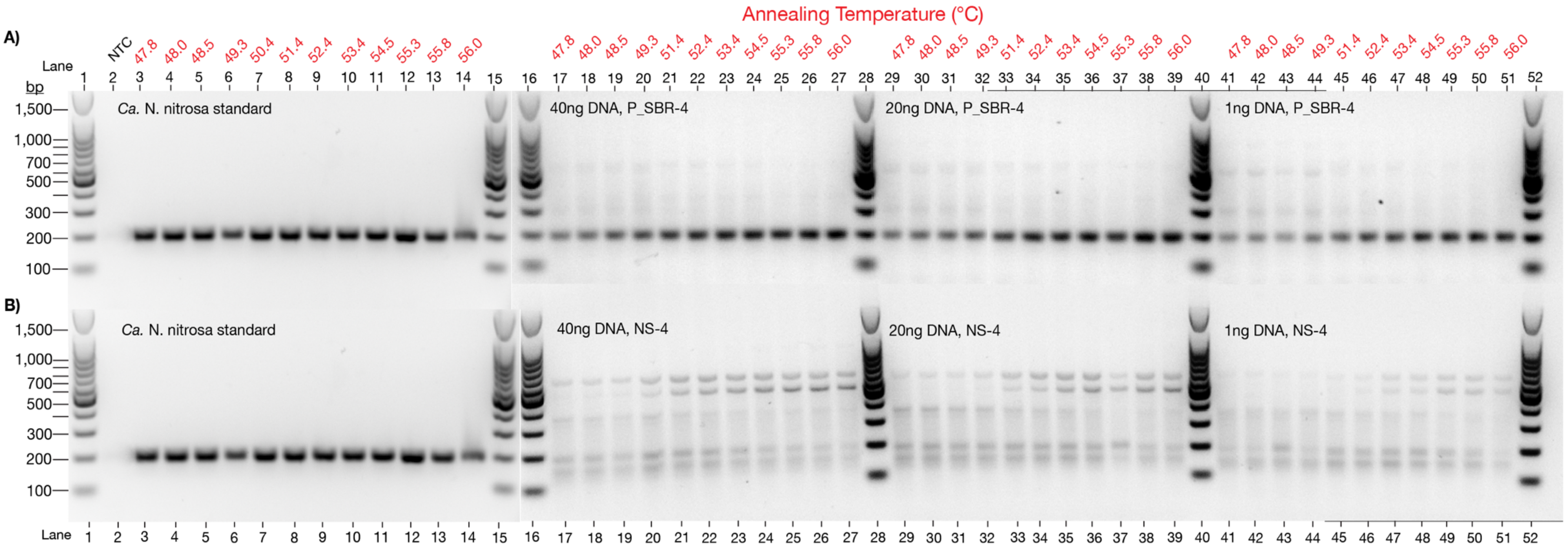

Figure S7. Total comammox (Ntsp-amoA 162F/359R) gradient PCR assay results with *Ca.* N. nitrosa standard and two bioreactor samples (A) P_SBR-4 and (B) NS-4, each diluted to 40ng DNA/reaction, 20ng DNA/reaction, and 1ng DNA/reaction. The gradient PCR was performed with annealing temperatures ranging between 47.8 °C and 56.0 °C, where 48 °C is the original design annealing temperature. The expected amplicon size is 198 bp for this assay. Off-target products are observed at approximately 300 bp and 600 bp in the P_SBR-4 sample and at approximately 150 bp, 350 bp, 600 bp and 700 bp in the NS-4 sample, and appear to be independent of the total DNA concentrations tested in the reaction.

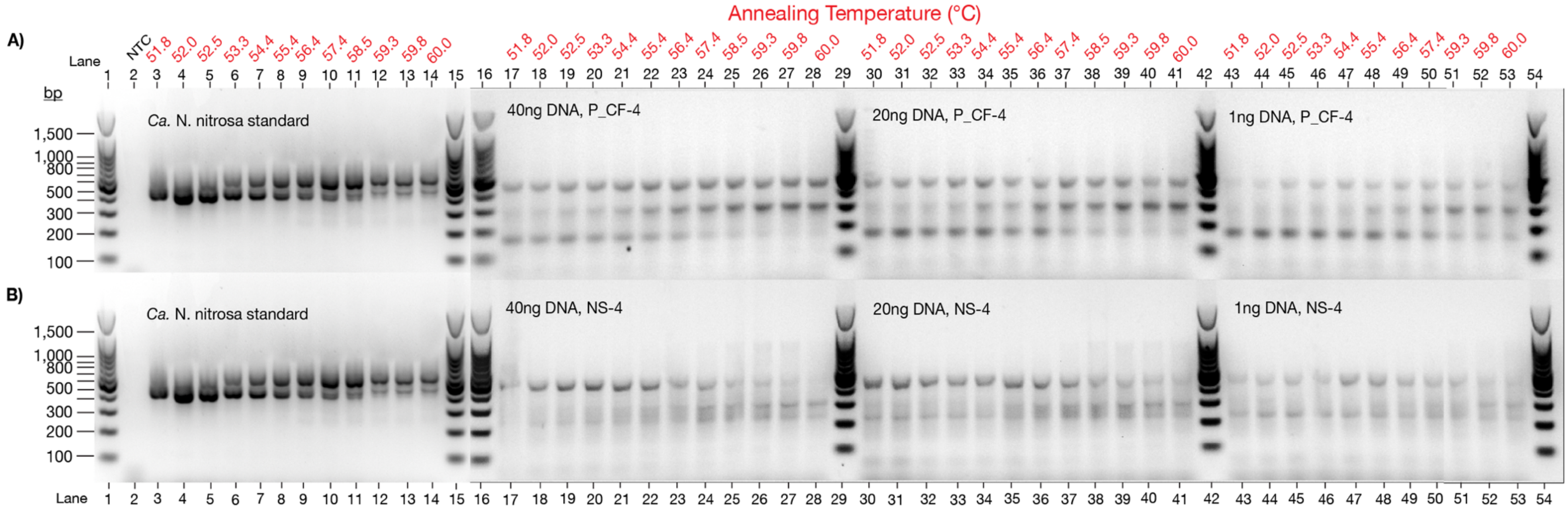

Figure S8. Clade A comammox (comaA-244f/659r) gradient PCR assay results with *Ca.* N. nitrosa standard and two bioreactor samples (A) P_CF-4 and (B) NS-4, each diluted to 40ng DNA/reaction, 20ng DNA/reaction, and 1ng DNA/reaction. The gradient PCR was performed with annealing temperatures ranging between 51.8 °C and 60.0 °C, where 52 °C is the original design annealing temperature. The expected amplicon size for this assay is 415 bp. Off-target products are observed at approximately 150 bp and 300 bp in the P_CF-4 sample and at approximately 250 bp and 300 bp in the NS-4 sample, and the amplification of these off-target products appear to be independent of the total DNA concentrations tested in the reaction. That is, with the exception of the P_CF-4 sample at the lowest total DNA concentration tested, where the 150 bp off-target product appears brighter than the target at the design annealing temperature, indicating the off-target product may be selectively amplified over the target in these conditions.

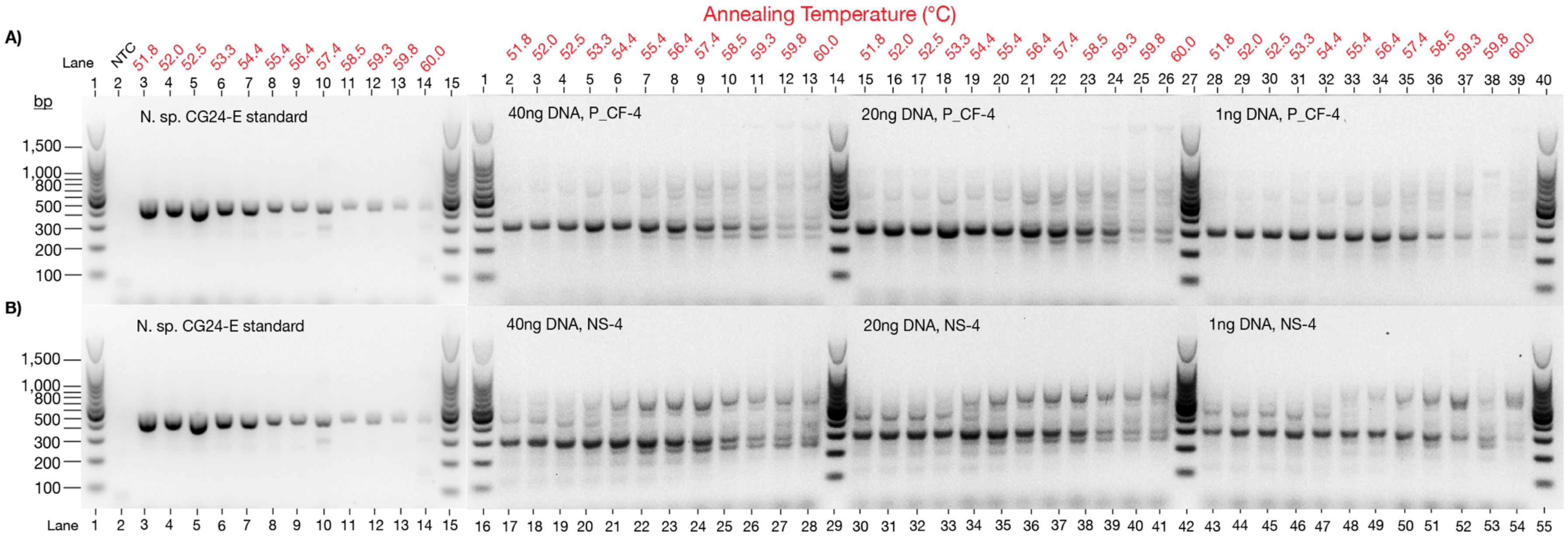

Figure S9. Clade B comammox (comaB-244f/659r) gradient PCR assay results with Nitrospira sp. CG-24E standard and two bioreactor samples (A) P_CF-4 and (B) NS-4, each diluted to 40ng DNA/reaction, 20ng DNA/reaction, and 1ng DNA/reaction. The gradient PCR was performed with annealing temperatures ranging between 51.8 °C and 60.0 °C, where 52 °C is the original design annealing temperature. The expected amplicon size for this assay is 415 bp. Multiple off-target products, especially one at 300 bp, appear at nearly every DNA concentration and temperature tested.

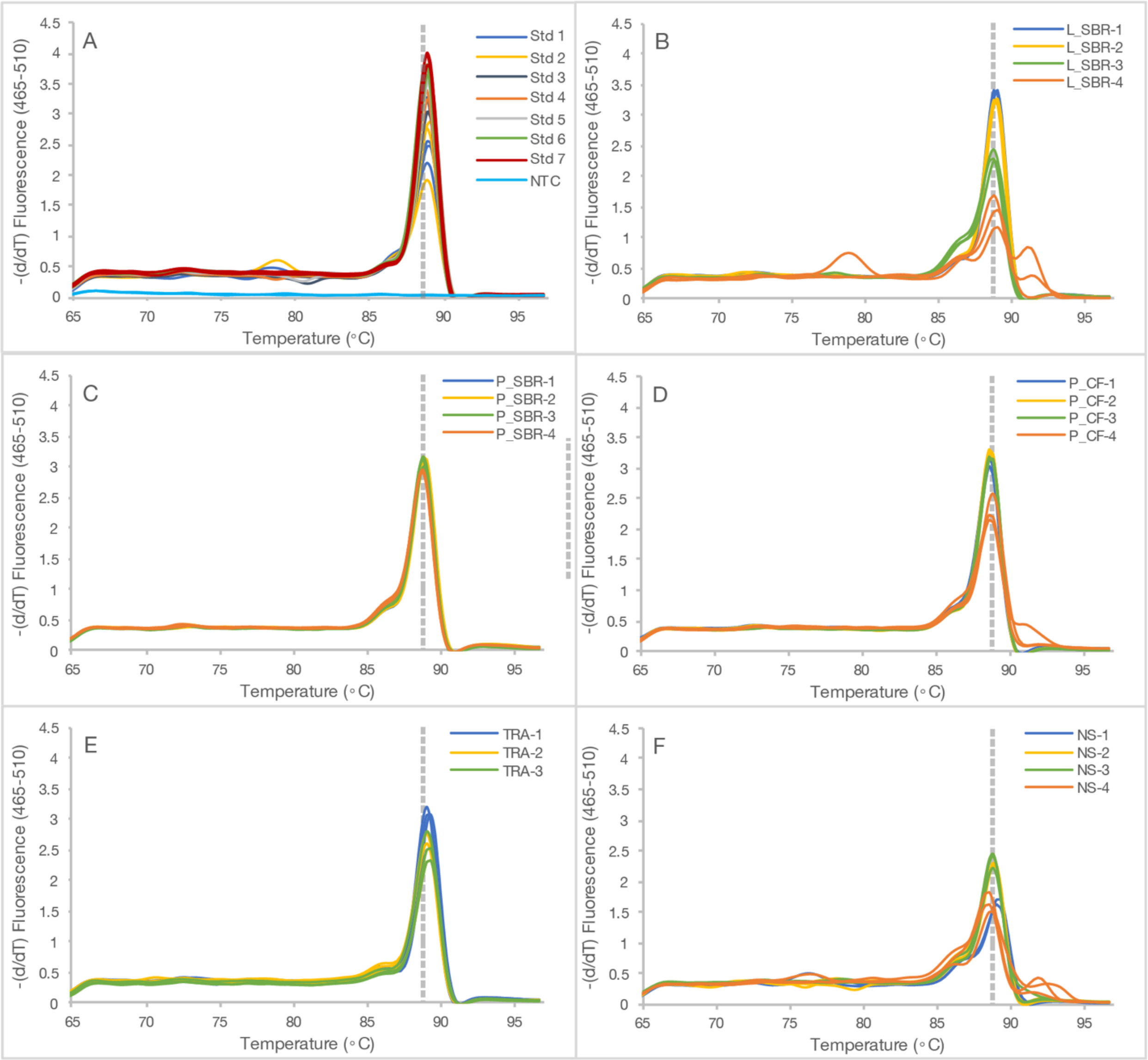

Figure S10. Comammox *Ca.* N. nitrosa assay (Nitrosa amoA-469F/812R) melting peaks after 45 cycles for A) N. nitrosa *amoA* standard curve*,* B) L-SBR samples, C) P-SBR samples, D) P-CF samples, E) TRA samples, and F) NS samples. The dashed lines in all panels represent the melting temperature for the standard. Triplicate data series are shown for each sample.

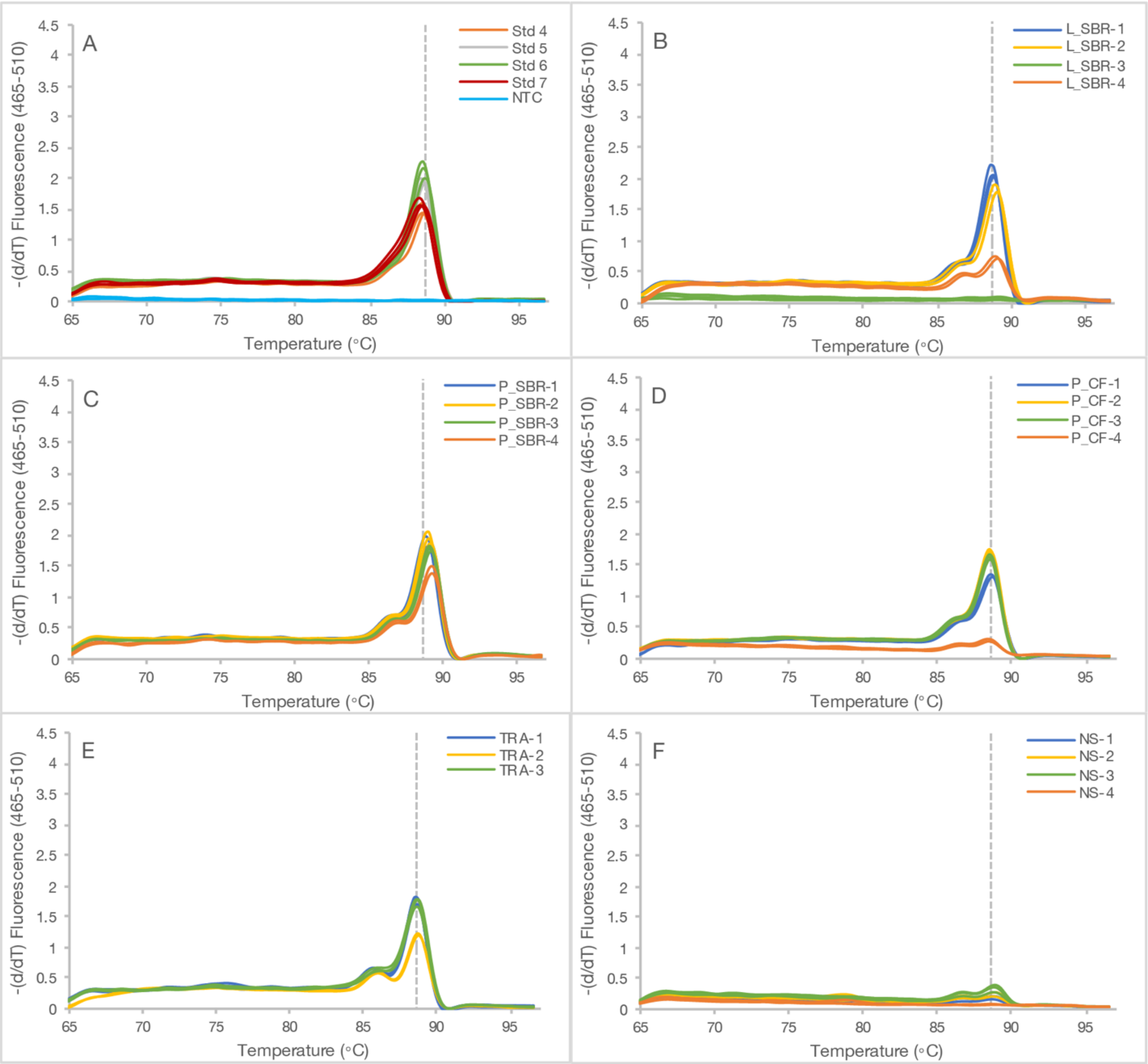

Figure S11. Comammox *Ca.* N. nitrosa assay (Nitrosa amoA-469F/812R) melting peaks after 30 cycles for A) N. nitrosa *amoA* standard curve*,* B) L-SBR samples, C) P-SBR samples, D) P-CF samples, E) TRA samples, and F) NS samples. The dashed lines in all panels represent the melting temperature for the standard. Triplicate data series are shown for each sample.

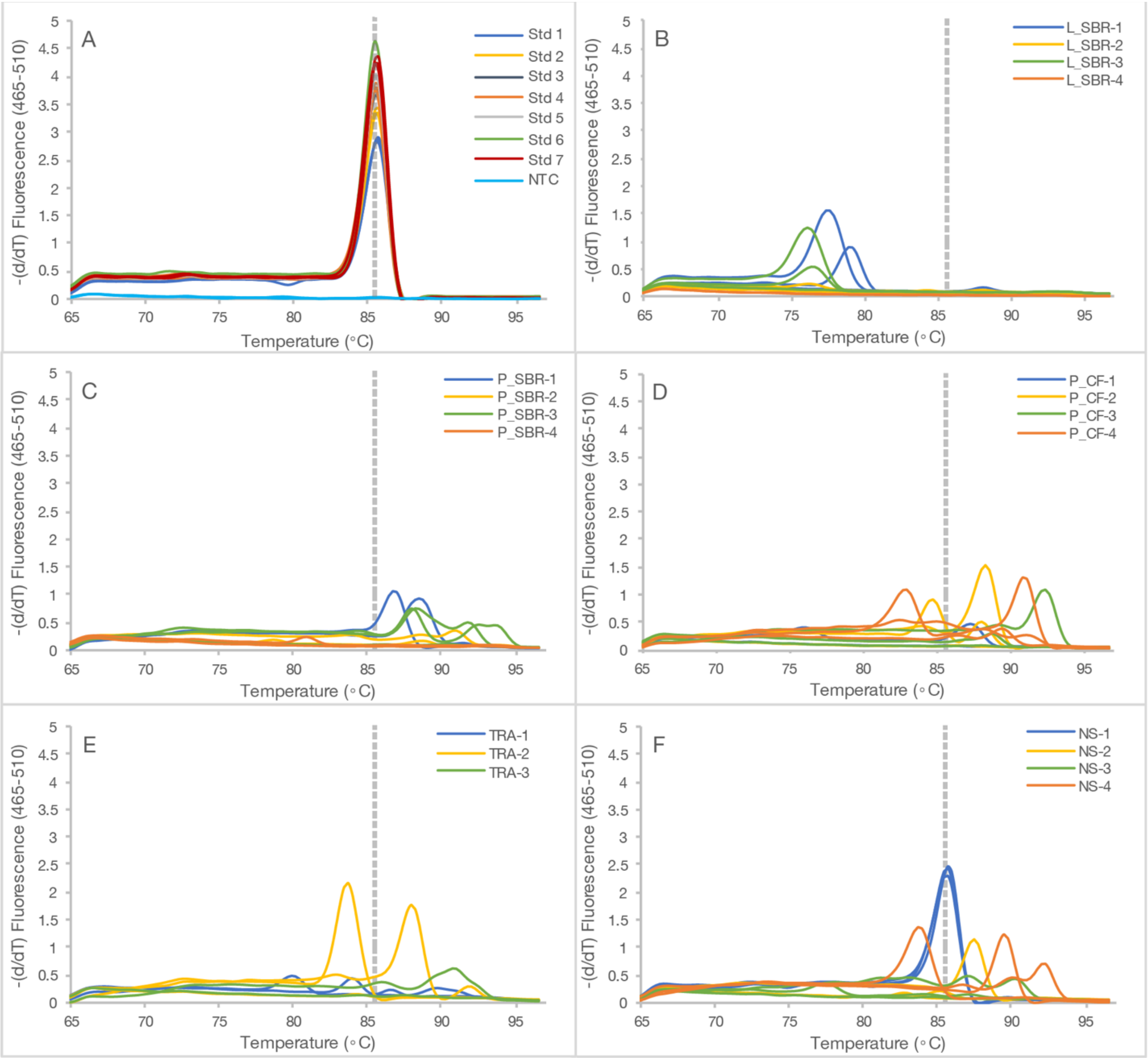

Figure S12. Comammox *Ca.* N. inopinata assay (Inopinata amoA-410F/815R) melting peaks after 45 cycles for A) N. inopinata *amoA* standard curve, B) L-SBR samples, C) P-SBR samples, D) P-CF samples, E) TRA samples, and F) NS samples. The dashed lines in all panels represent the melting temperatures for the standard. Triplicate data series are shown for each sample.

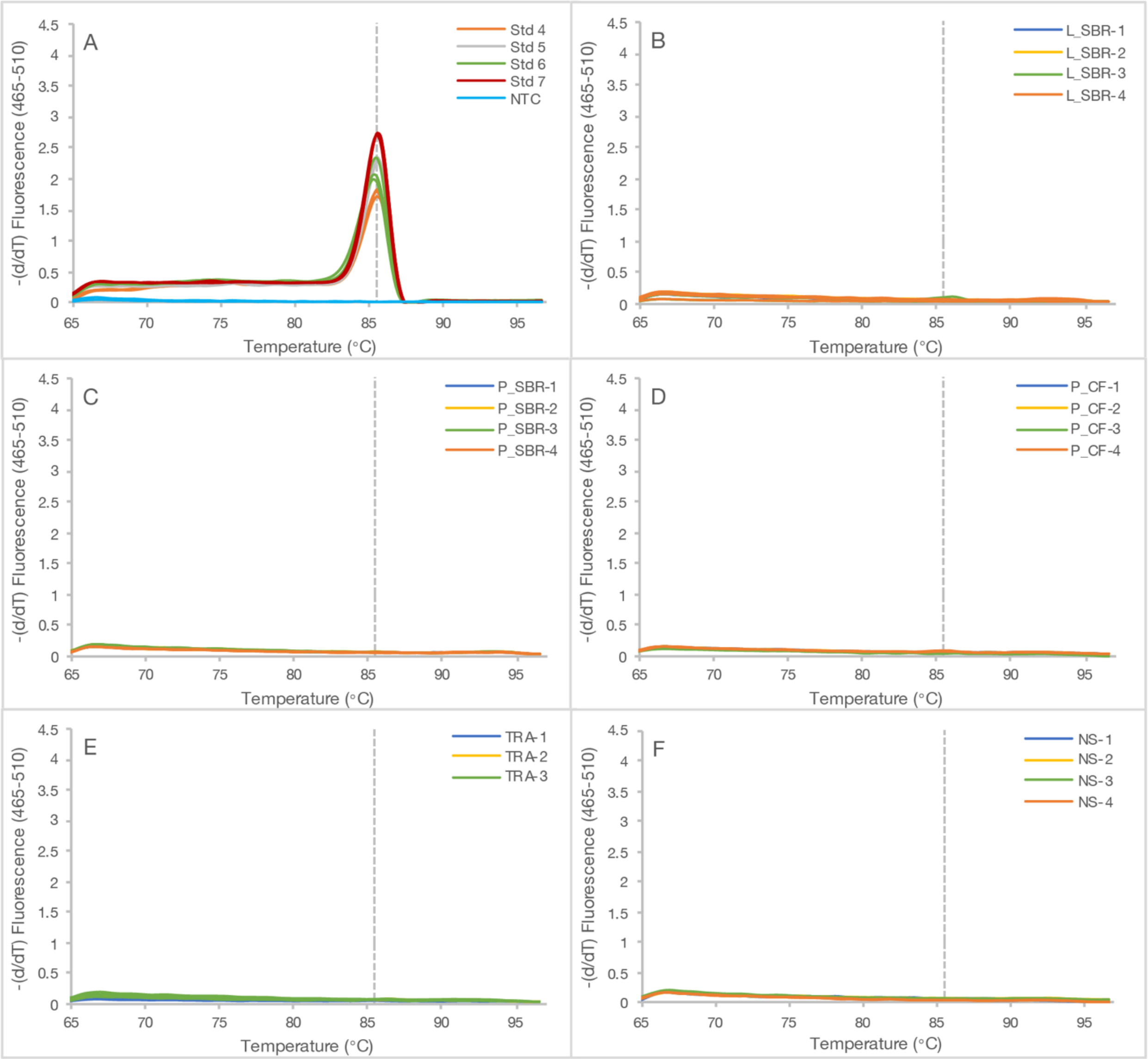

Figure S13. Comammox *Ca.* N. inopinata assay (Inopinata amoA-410F/815R) melting peaks after 30 cycles for A) N. inopinata *amoA* standard curve*,* B) L-SBR samples, C) P-SBR samples, D) P-CF samples, E) TRA samples, and F) NS samples. The dashed lines in all panels represent the melting temperature for the standard. Triplicate data series are shown for each sample.

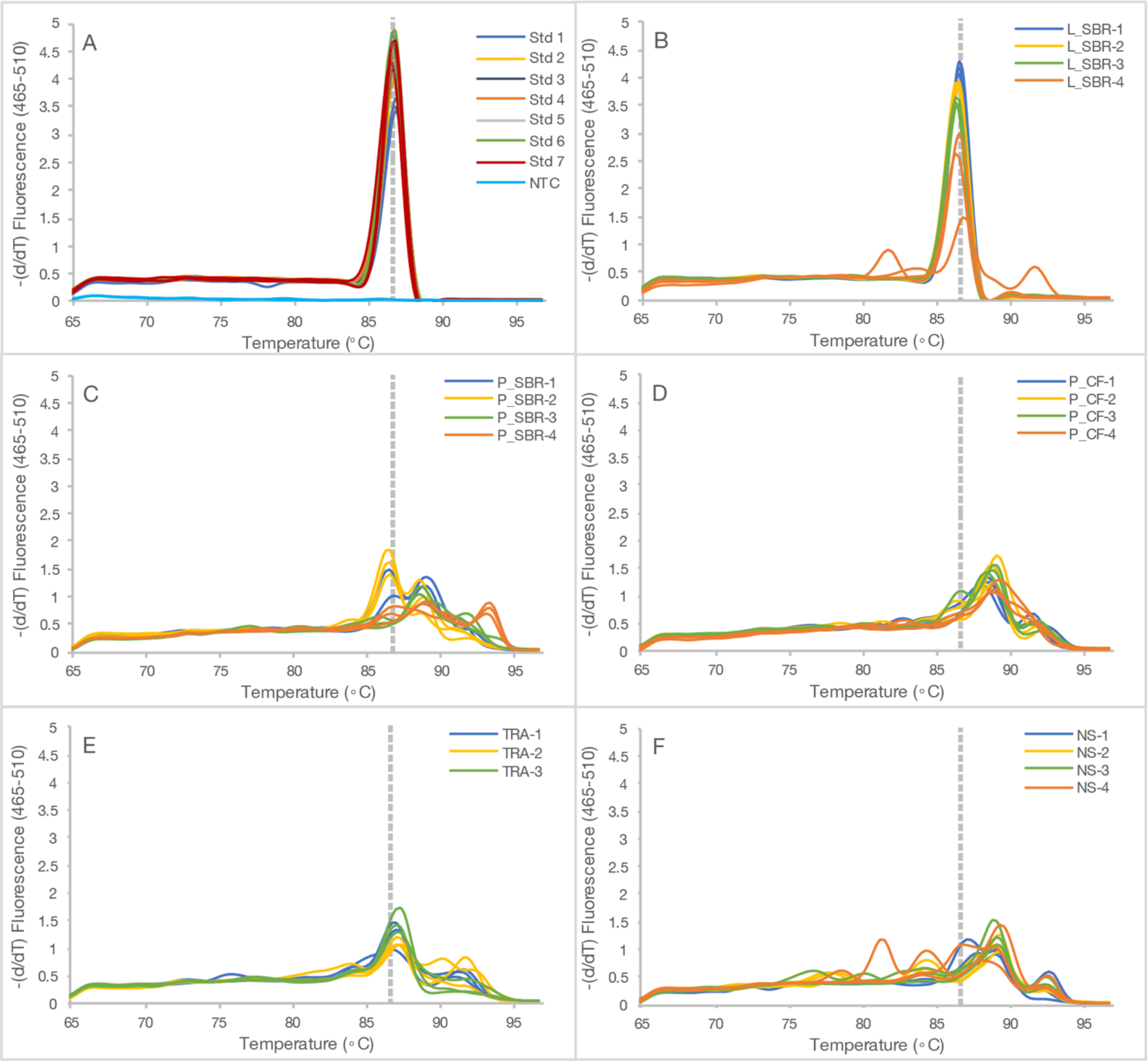

Figure S14. Comammox N. nitrificans assay (Nitrificans amoA-463F/836R) melting peaks after 45 cycles for A) N. nitrificans *amoA* standard curve, B) L-SBR samples, C) P-SBR samples, D) P-CF samples, E) TRA samples, and F) NS samples. The dashed lines in all panels represent the melting temperatures for the standard. Triplicate data series are shown for each sample.

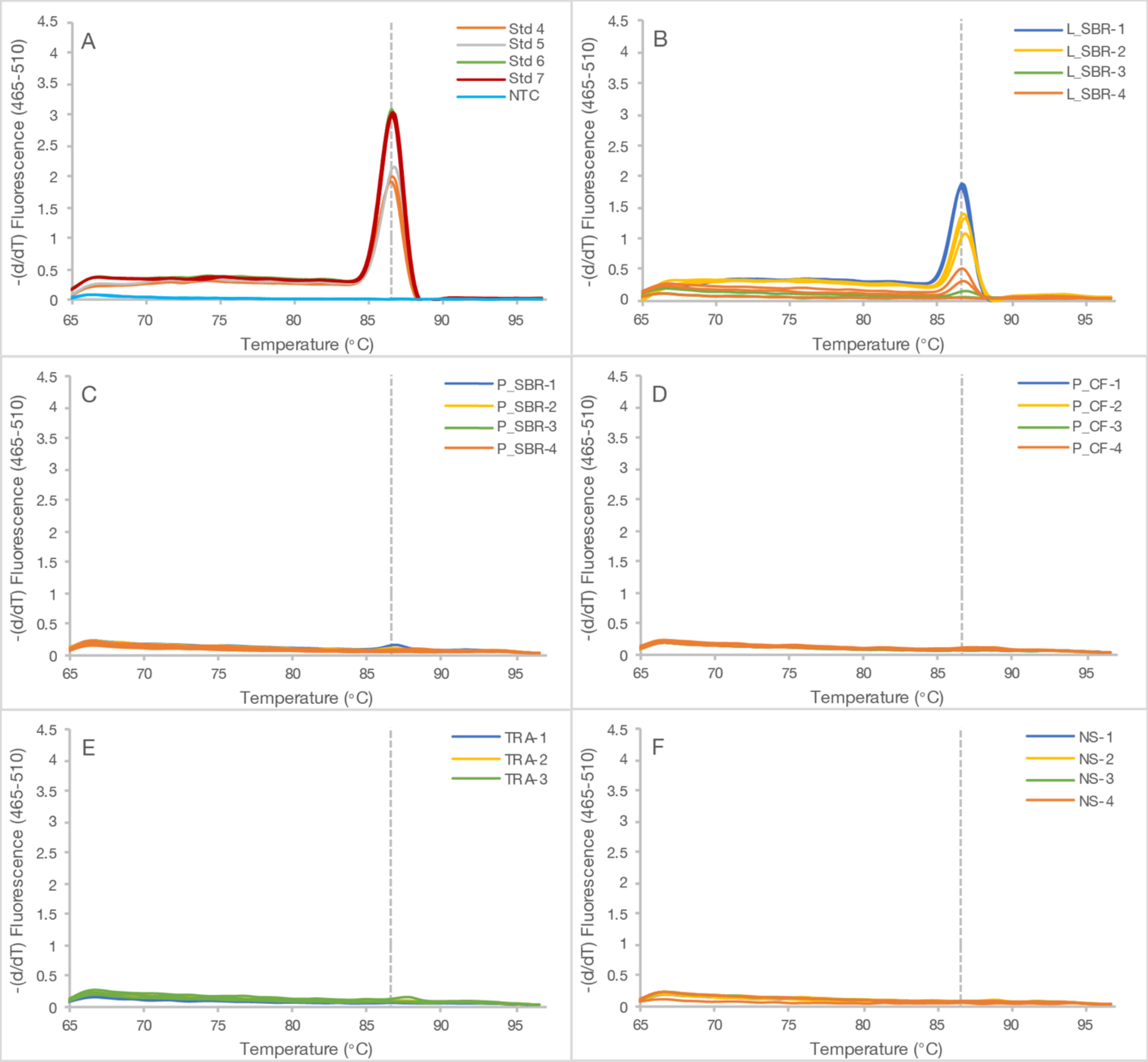

Figure S15. Comammox N. nitrificans assay (Nitrificans amoA-463F/836R) melting peaks after 30 cycles for A) N. nitrificans *amoA* standard curve*,* B) L-SBR samples, C) P-SBR samples, D) P-CF samples, E) TRA samples, and F) NS samples. The dashed lines in all panels represent the melting temperature for the standard. Triplicate data series are shown for each sample.

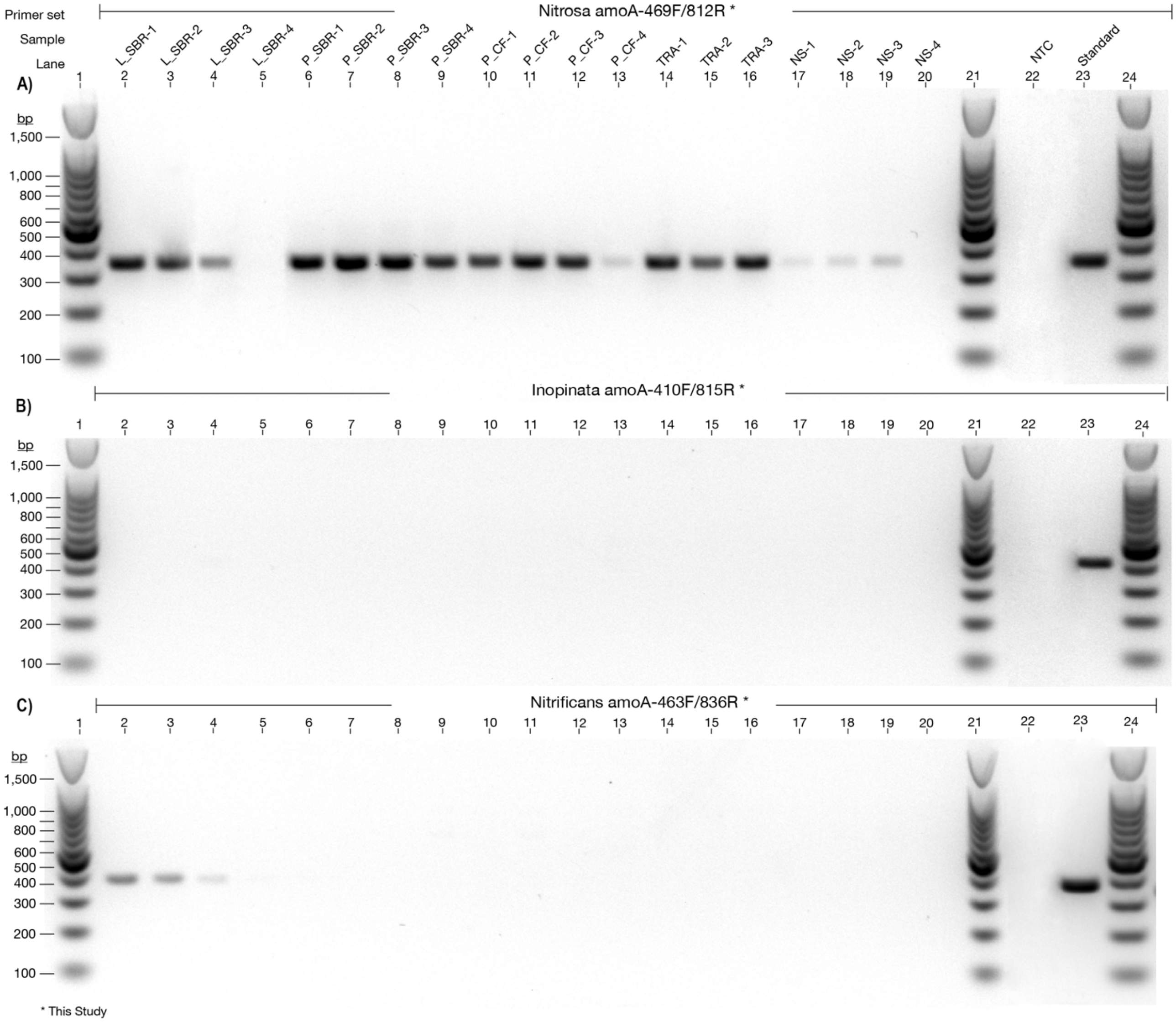

Figure S16. A qPCR assay with 30 cycles was generated to test each new primer set targeting A) *Ca.* N. nitrosa*,* B) N. inopinata*,* and C) N. nitrificans in one DNA sample from each bioreactor (lanes 2-20) with its corresponding non-template control (NTC, lane 22) and standard (lane 23). The expected band was present with *amoA* template from each external standard and some bioreactor samples. No off-target products were observed in the non-template control (NTC), bioreactor samples, or standards. Lanes 1, 21, and 24 correspond to 100 bp DNA size marker.

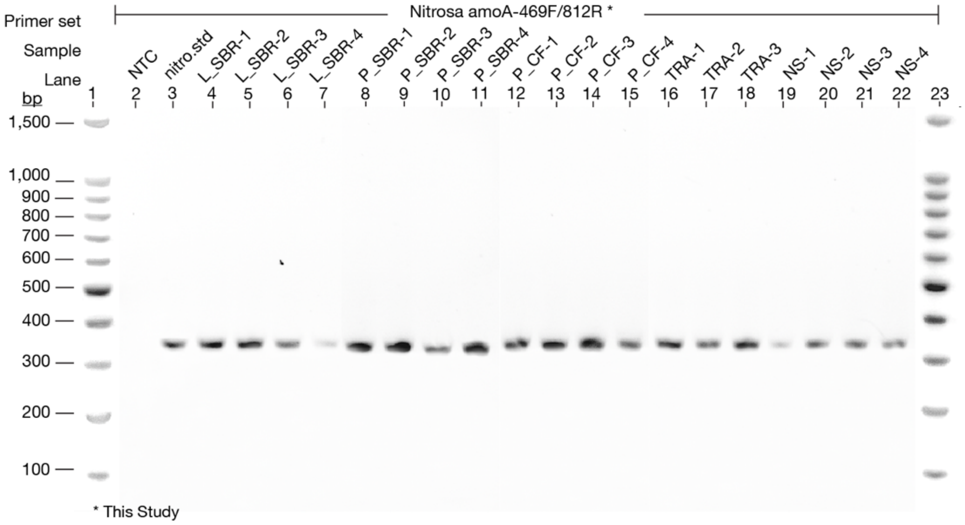

Figure S17. Agarose gel electrophoresis of qPCR products amplified after 45 cycles with the primers-set designed in this study targeting the *amoA* gene of N. nitrosa. The first and last lanes correspond to 100 bp DNA size marker.

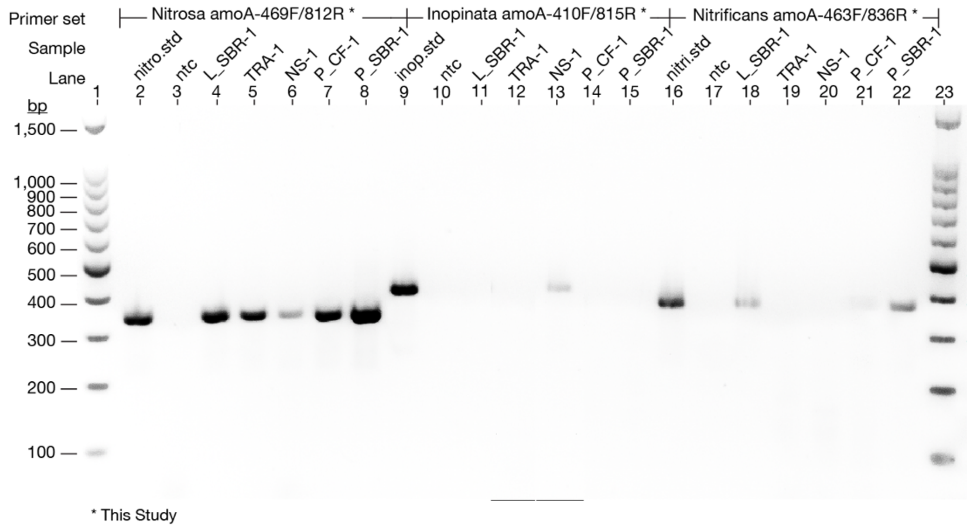

Figure S18. A PCR assay was generated to test each new primer set targeting *Ca.* N. nitrosa*,* N. inopinata*, and* N. nitrificans with its corresponding standard, a non-template control, and one DNA sample from each bioreactor. The expected band was present with *amoA* template from each external standard (*Ca.* N. nitrosa (nitro.), *Ca.* N. inopinata (inop.), and *Ca.* N. nitrificans (nitrif.)) and some bioreactor samples. No off-target products were observed in the non-template control (ntc), bioreactor samples, or standards. The first and last lanes correspond to 100 bp DNA size marker.

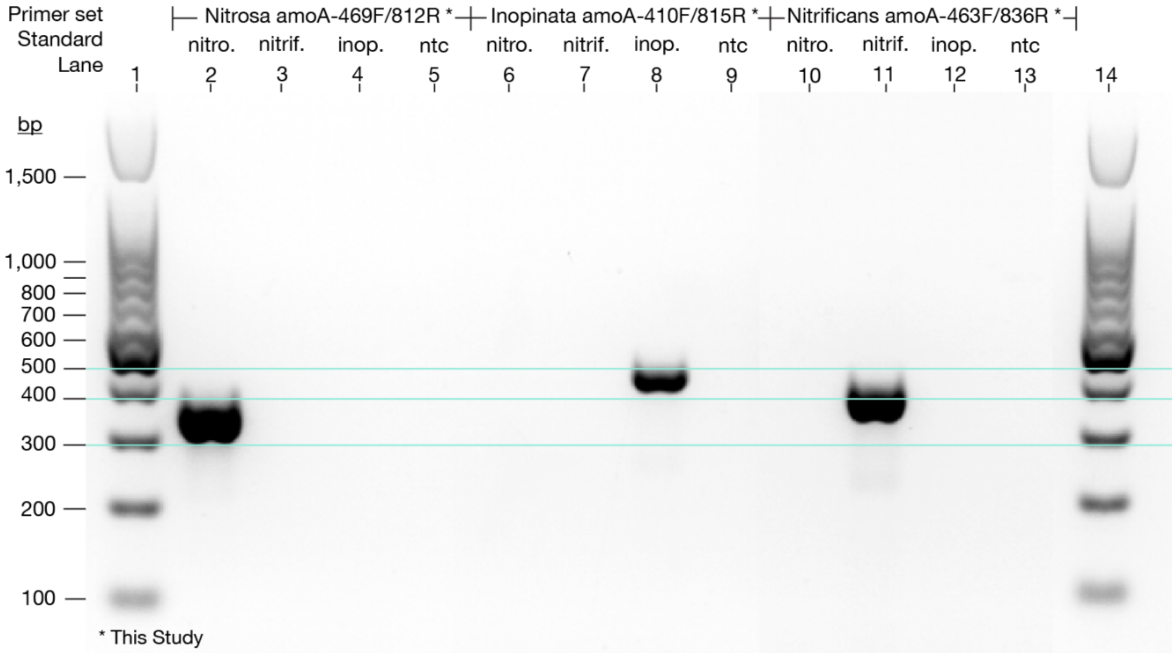

Figure S19. Cross-hybridization and primer dimers were not observed for the newly designed primer sets when combined in reaction with *amoA* template from each external standard (*Ca.* N. nitrosa (nitro.), *Ca.* N. inopinata (inop.), and *Ca.* N. nitrificans (nitrif.)) and non-template control (ntc). The first and last lanes correspond to 100 bp DNA size marker.

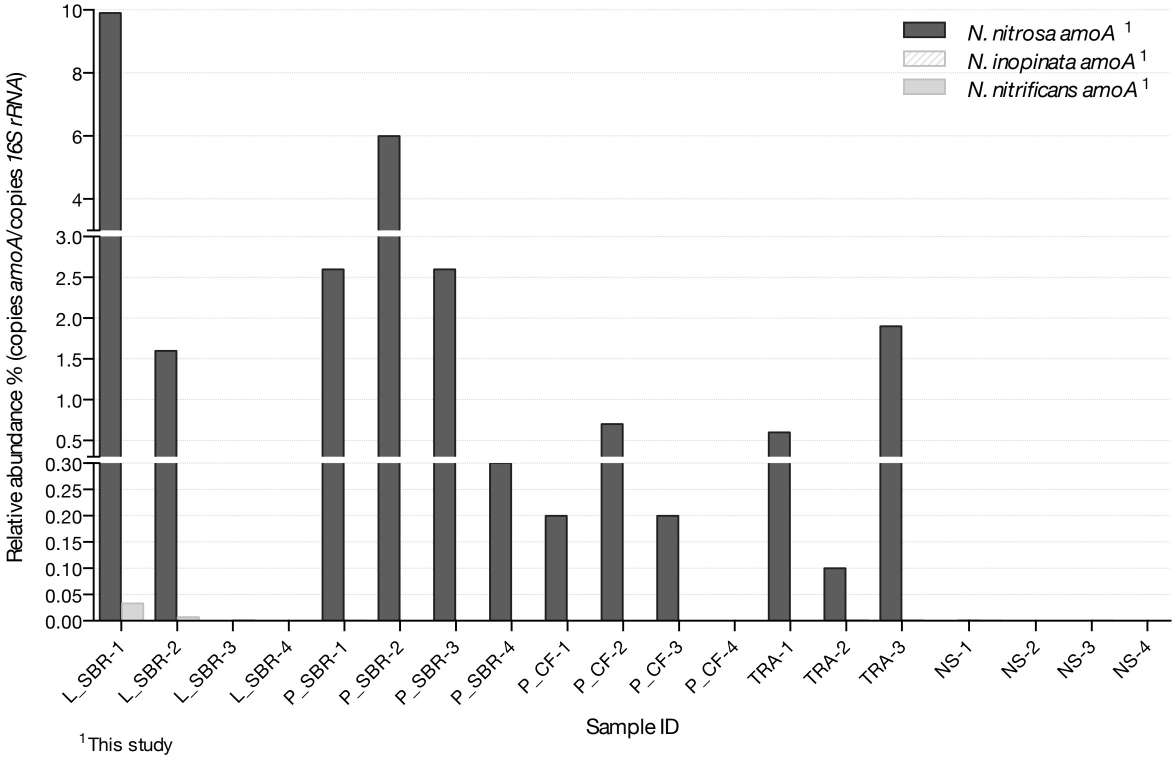

Figure S20. Percent relative abundance of comammox *Ca.* Nitrospira *amoA* genes to total bacteria (*amoA* gene copies/*16S rRNA* gene copies). Note the y-axis is not linear and is separated into three sections to show relative abundances below 0.30%, between 0.30 and 3.0%, and between 3.0 to 10%.

1.2 Supplementary Tables

Table S1. qPCR standard curves: slope, y-intercept, correlation coefficient, and PCR efficiency calculated from the approximate linear range for each assay included in this study.

	Primer set	Loq$_{10}$ linear range (log(SQ))*	Std curve slope (C_q/log(SQ))*	Std curve Y-intercept (C_q)*	Std curve correlation coefficient (R)	PCR efficiency (%)
**Comammox *Nitrospira* clade A**	**Standard template:** *Ca.* N. nitrosa ***amoA***					
	Nitrosa amoA-469F/812R[a]	**1 - 7**	-3.44 ± 0.07	37.6 ± 0.56	0.998 ± 0.001	96 ± 3.0
	Ntsp-amoA 162F/359R[b]	**3 - 7**	-3.55 ± 0.07	38.5 ± 0.43	0.999 ± 0.001	91 ± 2.6
	comaA-244f/659r[c]	**2 - 7**	-3.49 ± 0.07	37.0 ± 0.49	0.998 ± 0.001	95 ± 2.7
	**Standard template:** *Ca.* N. inopinata ***amoA***					
	Inopinata amoA-410F/815R[a]	**1- 7**	-3.63 ± 0.13	38.0 ± 0.65	0.997 ± 0.002	90 ± 3.0
	Ntsp-amoA 162F/359R[b]	**2 - 7**	-3.84 ± 0.12	39.5 ± 0.64	0.998 ± 0.002	82 ± 3.4
	comaA-244f/659r[c]	**3- 7**	-3.70 ± 0.08	37.3 ± 0.45	0.999 ± 0.001	86 ± 2.5
	**Standard template:** *Ca.* N. nitrificans ***amoA***					
	Nitrificans amoA-463F/836R[a]	**1 - 7**	-3.52 ± 0.07	37.8 ± 0.11	0.996 ± 0.004	92 ± 2.4
	Ntsp-amoA 162F/359R[b]	**2 - 7**	-3.43 ± 0.05	36.9 ± 0.27	0.999 ± 0.001	96 ± 1.8
	comaA-244f/659r[c]	**2 - 7**	-3.51 ± 0.08	36.2 ± 0.56	0.996 ± 0.006	93 ± 2.9
**Comammox *Nitrospira* clade B**	**Standard template:** *N. CG24_E* ***amoA***					
	comaB-244f/659r[c]	**1 - 7**	-3.76 ± 0.25	38.5 ± 0.23	0.992 ± 0.005	88 ± 0.6
**Total *16S rRNA***	**Standard template:** *Actinobacteria* (ac1) ***16S rRNA***					
	16S-341f/785r[d]	**2 - 7**	-3.88 ± 0.03	39.4 ± 0.15	0.997 ± 0.001	81 ± 0.8

*Where C_q = quantification cycle; SQ = starting quantity in copies per reaction; Std = standard
a. This study
b. Fowler et al., 2017
c. Pjevac et al., 2017
d. Klindworth et al., 2013; Thijs et al., 2017

Table S2. Total comammox primer mismatch table with comammox standard template *amoA* used in this study

		Forward Target Sequence																				
**Ntsp-amoA 162F**	5'	**G**	**G**	**A**	**T**	**T**	**T**	**C**	**T**	**G**	**G**	**N**	**T**	**S**	**G**	**A**	**T**	**T**	**G**	**G**	**A**	5'
Ca. N. nitrosa *amoA*1 (GCA_001458735.1)	3'	.	.	.	**C**	.	.	.	.	.	.	.	.	.	.	.	**C**	.	.	.	.	5'
Ca. N. nitrosa *amoA*2 (GCA_001458735.1)	3'	.	.	.	.	.	.	**T**	.	.	.	.	.	.	.	.	**C**	.	.	.	.	5'
Ca. N. inopinata *amoA* (GCA_001458695.1)	3'	.	.	.	.	.	.	.	.	.	.	.	.	.	.	.	.	.	.	.	.	5'
Ca. N. nitrificans *amoA* (GCA_001458775.1)	3'	.	.	.	.	.	.	.	.	.	.	.	.	.	.	.	.	.	.	.	.	5'

		Reverse Target Sequence																				
**Ntsp-amoA 359R**	5'	**W**	**A**	**G**	**T**	**T**	**N**	**G**	**A**	**C**	**C**	**A**	**C**	**C**	**A**	**S**	**T**	**A**	**C**	**C**	**A**	3'
Ca. N. nitrosa *amoA*1 (GCA_001458735.1)	3'	.	.	**T**	.	.	.	.	.	.	.	.	.	.	.	.	**G**	.	.	.	.	5'
Ca. N. nitrosa *amoA*2 (GCA_001458735.1)	3'	.	.	**T**	.	.	.	.	.	.	.	.	.	.	.	.	**G**	.	.	.	.	5'
Ca. N. inopinata *amoA* (GCA_001458695.1)	3'	.	.	.	.	.	.	.	.	.	.	.	.	.	.	.	**G**	.	.	.	.	5'
Ca. N. nitrificans *amoA* (GCA_001458775.1)	3'	.	.	**T**	.	.	.	.	.	.	.	.	.	.	.	.	**G**	.	.	.	.	5'

Table S3. Total comammox qPCR assay decision matrix summarizing criteria used to determine which samples were a non-detect, true positive, overestimate, or false positive

Total commamox assay[a]				
	Non-detect	**True Positive**	Overestimate	*False Positive*
Target band in gel?	No	**Yes**	Yes	*No*
Off target bands?	No	**No**	Yes	*-*
SQ > LOD *	No	**Yes**	Yes	*Yes*
SQ > LOQ *	No	**Yes or No**	Yes	*Yes or No*

		Qualitative		Quantitative			
Bioreactor Sample ID	Date	Target band in gel?	Off target bands?	Average C_q*	SQ > LOD*	SQ > LOQ*	Average T_m* within limits?
L_SBR-1	3/7/13	Yes	No	20	Yes	Yes	Yes
L_SBR-2	3/18/13	Yes	No	21	Yes	Yes	Yes
L_SBR-3	*4/17/13*	*No*	*Yes*	*24*	*Yes*	*Yes*	*Yes*
L_SBR-4	*10/3/14*	*No*	*No*	*22*	*Yes*	*Yes*	*No*
P_SBR-1	9/10/15	Yes	No	19	Yes	Yes	Yes
P_SBR-2	11/10/15	Yes	No	18	Yes	Yes	Yes
P_SBR-3	12/15/15	Yes	No	18	Yes	Yes	Yes
P_SBR-4	4/25/16	Yes	Yes	18	Yes	Yes	No
P_CF-1	9/9/15	Yes	No	24	Yes	Yes	Yes
P_CF-2	11/18/15	Yes	No	21	Yes	Yes	Yes
P_CF-3	12/30/15	Yes	Yes	22	Yes	Yes	Yes
P_CF-4	*4/26/16*	*No*	*Yes*	*20*	*Yes*	*Yes*	*Yes*
TRA-1	8/9/16	Yes	No	21	Yes	Yes	Yes
TRA-2	7/28/17	Yes	No	22	Yes	Yes	Yes
TRA-3	7/28/17	Yes	No	21	Yes	Yes	Yes
NS-1	9/9/15	Yes	Yes	22	Yes	Yes	No
NS-2	11/18/15	Yes	Yes	22	Yes	Yes	No
NS-3	12/30/15	Yes	Yes	22	Yes	Yes	Yes
NS-4	4/26/16	Yes	Yes	21	Yes	Yes	Yes

* C_q = quantification cycle, SQ = starting quantity, LOD = limit of detection, LOQ = limit of quantification, T_m = melting temperature

[a] Fowler et al. 2017

Table S4. Clade A comammox qPCR assay decision matrix summarizing criteria used to determine which samples were a non-detect, true positive, overestimate, or false positive

Clade A assay[b]				
	Non-detect	**True Positive**	Overestimate	*False Positive*
Target band in gel?	No	**Yes**	Yes	*No*
Off target bands?	No	**No**	Yes	*-*
SQ > LOD *	No	**Yes**	Yes	*Yes*
SQ > LOQ *	No	**Yes or No**	Yes	*Yes or No*

		Qualitative		Quantitative			
Reactor	Date	Target band in gel?	Off target bands?	Average C_q*	SQ > LOD*	SQ > LOQ*	Average T_m* within limits?
L_SBR-1	3/7/13	Yes	No	18	Yes	Yes	Yes
L_SBR-2	3/18/13	Yes	No	19	Yes	Yes	Yes
L_SBR-3	4/17/13	Yes	No	22	Yes	Yes	Yes
L_SBR-4	*10/3/14*	*No*	*Yes*	*22*	*Yes*	*Yes*	*No*
P_SBR-1	9/10/15	Yes	Yes	18	Yes	Yes	Yes
P_SBR-2	11/10/15	Yes	Yes	16	Yes	Yes	Yes
P_SBR-3	12/15/15	Yes	No	17	Yes	Yes	No
P_SBR-4	*4/25/16*	*No*	*Yes*	*19*	*Yes*	*Yes*	*No*
P_CF-1	*9/9/15*	*No*	*No*	*22*	*Yes*	*Yes*	*Yes*
P_CF-2	*11/18/15*	*No*	*Yes*	*19*	*Yes*	*Yes*	*No*
P_CF-3	*12/30/15*	*No*	*Yes*	*20*	*Yes*	*Yes*	*Yes*
P_CF-4	4/26/16	Yes	Yes	19	Yes	Yes	No
TRA-1	*8/9/16*	*No*	*Yes*	*21*	*Yes*	*Yes*	*Yes*
TRA-2	*7/28/17*	*No*	*Yes*	*22*	*Yes*	*Yes*	*No*
TRA-3	7/28/17	Yes	Yes	20	Yes	Yes	Yes
NS-1	9/9/15	Yes	Yes	22	Yes	Yes	Yes
NS-2	11/18/15	Yes	Yes	21	Yes	Yes	Yes
NS-3	12/30/15	Yes	Yes	20	Yes	Yes	Yes
NS-4	4/26/16	Yes	Yes	20	Yes	Yes	Yes

* C_q = quantification cycle, SQ = starting quantity, LOD = limit of detection, LOQ = limit of quantification, T_m = melting temperature
[b] Pjevac et al. 2017

Table S5. Clade B comammox qPCR assay decision matrix summarizing criteria used to determine which samples were a non-detect, true positive, overestimate, or false positive

Clade B assay[b]				
	Non-detect	**True Positive**	Overestimate	*False Positive*
Target band in gel?	No	**Yes**	Yes	*No*
Off target bands?	No	**No**	Yes	*-*
SQ > LOD *	No	**Yes**	Yes	*Yes*
SQ > LOQ *	No	**Yes or No**	Yes	*Yes or No*

		Qualitative		Quantitative			
Reactor	Date	Target band in gel?	Off target bands?	Average C_q*	SQ > LOD*	SQ > LOQ*	Average T_m* within limits?
L_SBR-1	*3/7/13*	*No*	*Yes*	*22*	*Yes*	*Yes*	*No*
L_SBR-2	*3/18/13*	*No*	*Yes*	*23*	*Yes*	*Yes*	*No*
L_SBR-3	*4/17/13*	*No*	*No*	*23*	*Yes*	*Yes*	*No*
L_SBR-4	*10/3/14*	*No*	*Yes*	*23*	*Yes*	*Yes*	*No*
P_SBR-1	*9/10/15*	*No*	*Yes*	*19*	*Yes*	*Yes*	*Yes*
P_SBR-2	*11/10/15*	*No*	*Yes*	*19*	*Yes*	*Yes*	*Yes*
P_SBR-3	*12/15/15*	*No*	*Yes*	*19*	*Yes*	*Yes*	*No*
P_SBR-4	*4/25/16*	*No*	*Yes*	*19*	*Yes*	*Yes*	*No*
P_CF-1	*9/9/15*	*No*	*Yes*	*22*	*Yes*	*Yes*	*Yes*
P_CF-2	*11/18/15*	*No*	*Yes*	*20*	*Yes*	*Yes*	*Yes*
P_CF-3	*12/30/15*	*No*	*Yes*	*21*	*Yes*	*Yes*	*Yes*
P_CF-4	4/26/16	Questionable	Yes	19	Yes	Yes	No
TRA-1	*8/9/16*	*No*	*Yes*	*21*	*Yes*	*Yes*	*No*
TRA-2	*7/28/17*	*No*	*Yes*	*21*	*Yes*	*Yes*	*No*
TRA-3	*7/28/17*	*No*	*Yes*	*21*	*Yes*	*Yes*	*No*
NS-1	*9/9/15*	*No*	*Yes*	*23*	*Yes*	*Yes*	*No*
NS-2	*11/18/15*	*No*	*Yes*	*22*	*Yes*	*Yes*	*No*
NS-3	*12/30/15*	*No*	*No*	*22*	*Yes*	*Yes*	*No*
NS-4	*4/26/16*	*No*	*Yes*	*21*	*Yes*	*Yes*	*No*

* C_q = quantification cycle, SQ = starting quantity, LOD = limit of detection, LOQ = limit of quantification, T_m = melting temperature

[b] Pjevac et al. 2017

Table S6. *Ca.* N. nitrosa comammox qPCR assay decision matrix summarizing criteria used to determine which samples were a non-detect, true positive, overestimate, or false positive

N. nitrosa assay[c]				
	Non-detect	**True Positive**	Overestimate	*False Positive*
Target band in gel?	No	**Yes**	Yes	*No*
Off target bands?	No	**No**	Yes	*-*
SQ > LOD *	No	**Yes**	Yes	*Yes*
SQ > LOQ *	No	**Yes or No**	Yes	*Yes or No*

		Qualitative		Quantitative			
Reactor	Date	Target band in gel?	Off target bands?	Average C_q*	SQ > LOD*	SQ > LOQ*	Average T_m* within limits?
L_SBR-1	3/7/13	Yes	No	17	Yes	Yes	Yes
L_SBR-2	3/18/13	Yes	No	19	Yes	Yes	Yes
L_SBR-3	4/17/13	Yes	No	27	Yes	Yes	Yes
L_SBR-4	10/3/14	Yes, after 30 cycles	No	34	No	No	Yes
P_SBR-1	9/10/15	Yes	No	18	Yes	Yes	Yes
P_SBR-2	11/10/15	Yes	No	17	Yes	Yes	Yes
P_SBR-3	12/15/15	Yes	No	18	Yes	Yes	Yes
P_SBR-4	4/25/16	Yes	No	21	Yes	Yes	Yes
P_CF-1	9/9/15	Yes	No	24	Yes	Yes	Yes
P_CF-2	11/18/15	Yes	No	21	Yes	Yes	Yes
P_CF-3	12/30/15	Yes	No	22	Yes	Yes	Yes
P_CF-4	4/26/16	Yes	No	29	No	No	Yes
TRA-1	8/9/16	Yes	No	21	Yes	Yes	Yes
TRA-2	7/28/17	Yes	No	25	Yes	Yes	Yes
TRA-3	7/28/17	Yes	No	20	Yes	Yes	Yes
NS-1	9/9/15	Yes	No	29	No	No	Yes
NS-2	11/18/15	Yes	No	29	No	No	Yes
NS-3	12/30/15	Yes	No	29	No	No	Yes
NS-4	4/26/16	Yes, after 30 cycles	No	33	No	No	Yes

* C_q = quantification cycle, SQ = starting quantity, LOD = limit of detection, LOQ = limit of quantification, T_m = melting temperature
[c] This Study

Table S7. *Ca.* N. inopinata comammox qPCR assay decision matrix summarizing criteria used to determine which samples were a non-detect, true positive, overestimate, or false positive

N. inopinata assay[c]	Non-detect	True Positive	Overestimate	False Positive
Target band in gel?	No	Yes	Yes	No
Off target bands?	No	No	Yes	-
SQ > LOD *	No	Yes	Yes	Yes
SQ > LOQ *	No	Yes or No	Yes	Yes or No

		Qualitative		Quantitative			
Reactor	Date	Target band in gel?	Off target bands?	Average C_q*	SQ > LOD*	SQ > LOQ*	Average T_m* within limits?
L_SBR-1	3/7/13	No	No	39	No	No	No
L_SBR-2	3/18/13	No	No	40	No	No	No
L_SBR-3	4/17/13	No	No	40	No	No	No
L_SBR-4	10/3/14	No	No	34	No	No	No
P_SBR-1	9/10/15	No	No	38	No	No	No
P_SBR-2	11/10/15	No	No	40	No	No	No
P_SBR-3	12/15/15	No	No	40	No	No	No
P_SBR-4	4/25/16	No	No	38	No	No	No
P_CF-1	9/9/15	No	No	39	No	No	No
P_CF-2	11/18/15	No	No	39	No	No	No
P_CF-3	12/30/15	No	No	39	No	No	No
P_CF-4	4/26/16	No	No	37	No	No	No
TRA-1	8/9/16	No	No	38	No	No	No
TRA-2	7/28/17	No	No	39	No	No	No
TRA-3	7/28/17	No	No	39	No	No	No
NS-1	9/9/15	Yes, after 30 cycles	No	33	No	No	Yes
NS-2	11/18/15	No	No	40	No	No	No
NS-3	12/30/15	No	No	39	No	No	No
NS-4	4/26/16	No	No	39	No	No	No

* C_q = quantification cycle, SQ = starting quantity, LOD = limit of detection, LOQ = limit of quantification, T_m = melting temperature

[c] This Study

Table S8. *Ca.* N nitrificans comammox qPCR assay decision matrix summarizing criteria used to determine which samples were a non-detect, true positive, overestimate, or false positive

N. nitrificans assay[c]				
	Non-detect	**True Positive**	Overestimate	*False Positive*
Target band in gel?	No	**Yes**	Yes	*No*
Off target bands?	No	**No**	Yes	-
SQ > LOD *	No	**Yes**	Yes	*Yes*
SQ > LOQ *	No	**Yes or No**	Yes	*Yes or No*

		Qualitative		Quantitative			
Reactor	Date	Target band in gel?	Off target bands?	Average C_q*	SQ > LOD*	SQ > LOQ*	Average T_m* within limits?
L_SBR-1	3/7/13	Yes	No	25	Yes	Yes	Yes
L_SBR-2	3/18/13	Yes	No	27	Yes	Yes	Yes
L_SBR-3	4/17/13	Yes	No	29	Yes	No	Yes
L_SBR-4	10/3/14	Yes, after 30 cycles	No	35	No	No	Yes
P_SBR-1	9/10/15	Yes, after 30 cycles	No	30	Yes	No	No
P_SBR-2	11/10/15	No	No	31	No	No	No
P_SBR-3	12/15/15	No	No	31	No	No	No
P_SBR-4	4/25/16	No	No	31	No	No	No
P_CF-1	9/9/15	No	No	33	No	No	No
P_CF-2	11/18/15	No	No	32	No	No	No
P_CF-3	12/30/15	No	No	31	No	No	No
P_CF-4	4/26/16	No	No	32	No	No	No
TRA-1	8/9/16	No	No	32	No	No	No
TRA-2	7/28/17	No	No	31	No	No	No
TRA-3	7/28/17	No	No	31	No	No	No
NS-1	9/9/15	No	No	33	No	No	No
NS-2	11/18/15	No	No	33	No	No	No
NS-3	12/30/15	No	No	31	No	No	No
NS-4	4/26/16	No	No	32	No	No	No

* C_q = quantification cycle, SQ = starting quantity, LOD = limit of detection, LOQ = limit of quantification, T_m = melting temperature

[c] This Study
